# Ammonia induces amyloidogenesis in astrocytes by promoting amyloid precursor protein translocation into the endoplasmic reticulum

**DOI:** 10.1016/j.jbc.2022.101933

**Published:** 2022-04-12

**Authors:** Ayaka Komatsu, Izumi Iida, Yusuke Nasu, Genki Ito, Fumiko Harada, Sari Kishikawa, Stephen J. Moss, Takeyasu Maeda, Miho Terunuma

**Affiliations:** 1 Division of Oral Biochemistry, Faculty of Dentistry & Graduate School of Medical and Dental Sciences, Niigata University, Niigata, Japan; 2 Center for Advanced Oral Science, Faculty of Dentistry & Graduate School of Medical and Dental Sciences, Niigata University, Niigata, Japan; 3 Department of Neuroscience, Tufts University School of Medicine, Boston, Massachusetts, USA

**Keywords:** ammonia, astrocytes, amyloid beta, amyloid precursor protein, protein translocation, Aβ, amyloid beta, AD, Alzheimer’s disease, APP, amyloid precursor protein, AQP4, aquaporin-4, BACE1, beta-site APP cleaving enzyme 1, CHX, cycloheximide, ER, endoplasmic reticulum, GFAP, glial fibrillary acidic protein, imAPP, immature APP, IRE1, inositol-requiring enzyme 1, mAPP, mature amyloid precursor protein, MEM, minimal essential medium, NKCC1, (Na^+^, K^+^, 2Cl^-^ cotransporter), OASIS, old astrocyte specifically induced substance, PFA, paraformaldehyde, TAA, thioacetamide, TGN, *trans*-Golgi network

## Abstract

Hyperammonemia is known to cause various neurological dysfunctions such as seizures and cognitive impairment. Several studies have suggested that hyperammonemia may also be linked to the development of Alzheimer’s disease (AD). However, the direct evidence for a role of ammonia in the pathophysiology of AD remains to be discovered. Herein, we report that hyperammonemia increases the amount of mature amyloid precursor protein (mAPP) in astrocytes, the largest and most prevalent type of glial cells in the central nervous system that are capable of metabolizing glutamate and ammonia, and promotes amyloid beta (Aβ) production. We demonstrate the accumulation of mAPP in astrocytes was primarily due to enhanced endocytosis of mAPP from the plasma membrane. A large proportion of internalized mAPP was targeted not to the lysosome, but to the endoplasmic reticulum, where processing enzymes β-secretase BACE1 (beta-site APP cleaving enzyme 1) and γ-secretase presenilin-1 are expressed, and mAPP is cleaved to produce Aβ. Finally, we show the ammonia-induced production of Aβ in astrocytic endoplasmic reticulum was specific to Aβ42, a principal component of senile plaques in AD patients. Our studies uncover a novel mechanism of Aβ42 production in astrocytes and also provide the first evidence that ammonia induces the pathogenesis of AD by regulating astrocyte function.

Ammonia is a potent neurotoxin that causes severe damage to the central nervous system. It is formed in nearly all tissues of the vertebrate organism and is a byproduct of cellular metabolism: hydrolysis of amide groups of proteins, degradation of amino acids, deamination of amino-purines and -pyrimidines, oxidative deamination of primary amines, and glycine catabolism (1). Deficient hepatic urea formation, urea cycle failure, and bacterial infection in the gut are the major causes of pathological accumulation of ammonia, which results in hyperammonemia (2, 3). Hyperammonemia has been shown to be a key pathogenic feature of the neuropsychiatric disorder hepatic encephalopathy (HE), which leads to an alteration in mental status and coma, as well as various neurological dysfunctions, such as tremor, ataxia, and seizure (4, 5, 6). Since ammonia is a neurotoxic agent, removal of excessive ammonia from the blood is critical for maintaining brain health. In the brain, where the urea cycle does not occur, astrocytes, the most prevalent glial cells in the brain, detoxify ammonia *via* glutamine synthetase and convert it to glutamine (7).

Previous research has suggested the existence of a correlation between ammonia and Alzheimer's disease (AD). Excessive formation of ammonia, as well as elevated blood ammonia concentrations, have been detected in the brains of AD patients (8, 9, 10, 11). Furthermore, research in AD patients has shown reduced activity of astrocytic glutamine synthetase and increased activity of adenosine monophosphate deaminase, which hydrolyzes AMP to inosine monophosphate and ammonia, suggesting an abnormal ammonia metabolism in the AD brain (12, 13). Taken together, all these findings indicate the contribution of ammonia in the symptoms of AD; however, direct evidence for a role of ammonia in the pathophysiology of AD is not concrete.

AD is the leading cause of neurodegenerative dementia, symptomatically characterized by cognitive decline, irreversible memory loss, disorientation, and language impairment. AD pathogenesis is widely believed to be driven by amyloid plaques composed primarily of aggregated amyloid beta (Aβ) peptides and neurofibrillary tangles of the microtubule-binding protein tau. In contrast to amyloid plaques, neurofibrillary tangles are less specific to AD, as they are seen in a greater variety of less common neurodegenerative diseases, such as progressive supranuclear palsy, corticobasal degeneration, and subtypes of frontotemporal dementia (14). Pathological, genetic, and biologic evidence have supported an important role for Aβ in the development of AD. An ∼40 amino acid Aβ peptide is derived from the amyloid precursor protein (APP). APP is a type I membrane protein with a large N-terminal extracellular domain, a single transmembrane domain, and a short cytoplasmic tail (15). Newly synthesized APP is subjected to N-glycosylation (immature APP: imAPP) in the endoplasmic reticulum (ER) and is subsequently subjected to O-glycosylation in the Golgi compartment as it reaches its mature form (16). Therefore, mature APP (mAPP) at the plasma membrane possesses both N- and O-glycans. APP is sequentially cleaved by two membrane-bound endoproteases, β- and γ-secretase, to generate Aβ. Numerous different Aβ species are known to exist, but Aβ40 are the most abundant peptides (∼90%) followed by Aβ42 (∼10%). In particular, Aβ42 is more hydrophobic and fibrillogenic than Aβ40 and is thought to be the principal species deposited in the AD brain (17).

There is compelling evidence that production of Aβ is closely associated with neuroinflammation, and reactive astrocytes are localized tightly around amyloid plaques (18). Astrocytes are known to engulf dead cells as well as protein aggregates such as Aβ and α-synuclein (19, 20, 21). In addition, astrocytes with high Aβ load are frequently found in AD brain tissue (22). Here, we show that ammonia directly triggers the production and accumulation of Aβ42 in astrocytes by inducing the endocytosis of mAPP from the plasma membrane, leading to its translocation to the ER. All of our findings provide evidence for a novel role of ammonia in the pathogenesis of AD, describing the direct connection between hyperammonemia and AD.

## Results

### Ammonia increases the expression of APP in primary cultured astrocytes

**Figure 1:**
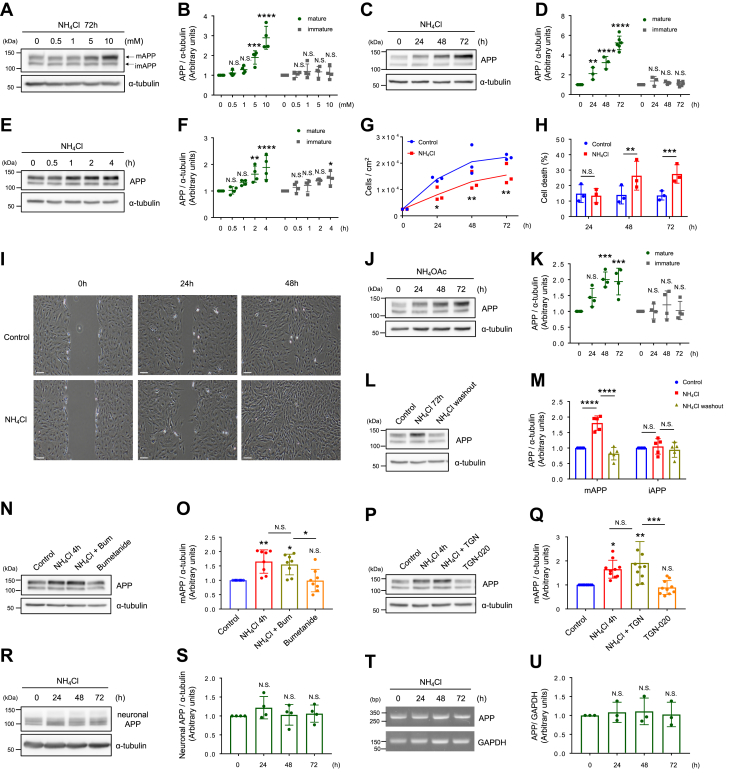
**Ammonia elevates the expression of amyloid precursor protein in astrocytes.***A*, representative western blots of APP and α-tubulin at various dose of NH_4_Cl treatments in cultured astrocytes. *B*, quantification of mature APP (*green*) and immature APP (*gray*). n = 4. Two-way ANOVA followed by Sidak's multiple comparisons test, ∗∗∗*p* < 0.001, ∗∗∗∗*p* < 0.0001. N.S. = not significant. *C*, representative western blots of APP and α-tubulin after 10 mM NH_4_Cl treatments in cultured astrocytes. *D*, quantification of mature APP (*green*) and immature APP (*gray*). 0 h, 72 h, n = 6; 24 h, 48 h, n = 3. Two-way ANOVA followed by Sidak's multiple comparisons test, ∗∗*p* < 0.01, ∗∗∗∗*p* < 0.0001. N.S. = not significant. *E*, representative western blots of APP and α-tubulin after 10 mM NH_4_Cl treatments in cultured astrocytes. *F*, quantification of mature APP (*green*) and immature APP (*gray*). n = 4. Two-way ANOVA followed by Sidak’s multiple comparisons test, ∗*p* < 0.05, ∗∗*p* < 0.01, ∗∗∗∗*p* < 0.0001. *G*, time-dependent proliferation of control (*blue*) and NH_4_Cl-treated (*red*) astrocytes. n = 3, two-way ANOVA followed by Sidak’s multiple comparison tests, ∗*p* < 0.05, ∗∗ *p* < 0.01. *H*, time course analysis of cell death induced by NH_4_Cl. Dead cells were counted and expressed as a percentage of dead cells from the total population. n = 3, two-way ANOVA followed by Sidak’s multiple comparison tests, ∗∗*p* < 0.01, ∗∗∗*p* < 0.001. N.S. = not significant. *I*, images of wound made in control and NH_4_Cl-treated astrocytes. n = 3. The scale bar represents 200 μm. *J*, representative western blots of APP and α-tubulin after 10 mM ammonium acetate (NH_4_OAc) treatments. *K*, quantification of mature APP (*green*) and immature APP (*gray*). n = 4. Two-way ANOVA followed by Sidak’s multiple comparisons test, ∗∗∗*p* < 0.001. N.S. = not significant. *L*, representative western blots of APP and α-tubulin before (NH_4_Cl 72 h) and after the removal of NH_4_Cl for 72 h (washout) in cultured astrocytes. *M*, quantification of mature APP (*green*) and immature APP (*gray*). n = 5. One-way ANOVA followed by Tukey's multiple comparisons test, ∗∗∗∗*p* < 0.0001. N.S. = not significant. *N*, representative western blots of APP and α-tubulin in the NH_4_Cl- (10 mM, 4 h) and bumetanide- (Bum; 75 μM) treated astrocytes. *O*, quantification of mature APP. n = 8. One-way ANOVA followed by Tukey's multiple comparisons test, ∗*p* < 0.05, ∗∗*p* < 0.01. N.S. = not significant. *P*, representative western blots of APP and α-tubulin in the NH_4_Cl- (10 mM, 4 h) and TGN-020- (TGN; 10 μM) treated astrocytes. *Q*, quantification of mature APP. n = 10. One-way ANOVA followed by Tukey's multiple comparisons test, ∗*p* < 0.05, ∗∗*p* < 0.01, ∗∗∗*p* < 0.001. N.S. = not significant. *R*, representative western blots of APP and α-tubulin after 10 mM NH_4_Cl treatments in cultured cortical neurons. *S*, quantification of neuronal APP. n = 4. One-way ANOVA followed by Dunnett’s multiple comparison test. N.S. = not significant. *T*, effect of NH_4_Cl on APP mRNA levels in cultured astrocytes. Representative image of RT-PCR of APP and GAPDH. *U*, bar graph represents the quantification of APP mRNA levels. Expression of target gene was normalized to that of GAPDH. n = 3, One-way ANOVA followed by Dunnett's multiple comparisons test. N.S. = not significant. APP, amyloid precursor protein; TGN, *N*-1,3,4-Thiadiazol-2-yl-3-pyridinecarboxamide.

To examine whether ammonia-treated astrocytes are amyloidogenic, we prepared cultured cortical astrocytes from rat E18 to 19 embryos (Fig. S1, *A*–*E*). We found that prolonged NH_4_Cl treatment significantly increased the amount of APP in dose- and time-dependent manner (Fig. 1, *A*–*D*). Interestingly, elevated APP expression was only detected in the mAPP (N- and O-glycosylated) and not in the imAPP (N-glycosylated). These substantial changes in mAPP expression were also observed after a short application of NH_4_Cl (Fig. 1, *E* and *F*). Of note, the mitotic activity of astrocytes was significantly suppressed by NH_4_Cl (Fig. 1, *G* and *H*); however, wounding of a monolayer of primary astrocytes leads to a slow but directed migration, and no difference in cell migration was observed in NH_4_Cl-treated astrocytes (Fig. 1*I*). We also examined if ammonium acetate, another type of ammonium salt, increases the level of APP in astrocytes. We observed similar changes in APP levels as those found in NH_4_Cl-treated cells (Fig. 1, *J* and *K*). Furthermore, we found that the removal of NH_4_Cl from culture medium reduced APP to baseline levels (Fig. 1, *L* and *M*).

A high level of NH_4_Cl inhibits protein degradation by increasing the pH of lysosomes (23). We found that the pH of cultured media containing 10 mM NH_4_Cl was around 7.9 (n = 7), whereas in control cultured media, the pH was around 7.5 (n = 7). The pH of culture media containing 10 mM ammonium acetate was around 7.5 (n = 4). We further examined if artificially elevated pH in the culture medium affects APP levels in astrocytes. We found that both short-term and long-term incubation of cultured astrocytes with alkaline medium (pH 8.7) did not alter APP levels (Fig. S2, *A*–*D*). In the brain, ammonia is the precursor of glutamine, a substrate for the production of both excitatory and inhibitory neurotransmitters (7). Thus, we wondered if elevated glutamine alters APP levels. We first removed glutamine from the culture medium for 36 h (glutamine starvation) and stimulated with 4 mM glutamine for up to 24 h (24). We found that glutamine does not regulate APP levels in astrocytes (Fig. S2, *E*–*F*). In addition, prolonged inhibition of glutamine synthesis with L-methionine sulfoximine had no effect on APP expression (Fig. S2, *G*–*H*). These results revealed that ammonia modulates astrocytic APP expression levels, an effect that is independent of pH. Several ammonia transporters such as the Na^+^, K^+^, 2Cl^−^ cotransporter (NKCC1) and aquaporin-4 (AQP4) have been reported in astrocytes (25, 26). Therefore, we used the NKCC1 inhibitor bumetanide and the AQP4 inhibitor TGN-020 and examined the expression of mAPP after NH_4_Cl stimulation (6, 27). Neither bumetanide nor TGN-020 prevented the NH_4_Cl-induced elevation of mAPP (Fig. 1, *N*–*Q*). These results suggested that mAPP levels in astrocytes were not regulated by these two ammonia transporters. In addition, we examined the time-dependent effect of NH_4_Cl in cultured cortical neurons and no change in APP expression was detected (Fig. 1, *R* and *S*). To determine if NH_4_Cl-induced elevated mAPP was caused by enhanced APP synthesis, we analyzed mRNA expression of APP. Prolonged treatment of cultured astrocytes with NH_4_Cl did not alter APP mRNA (Fig. 1, *T* and *U*).

### Astrocytic APP undergoes clathrin-mediated endocytosis and ammonia facilitates the rate of APP endocytosis

**Figure 2:**
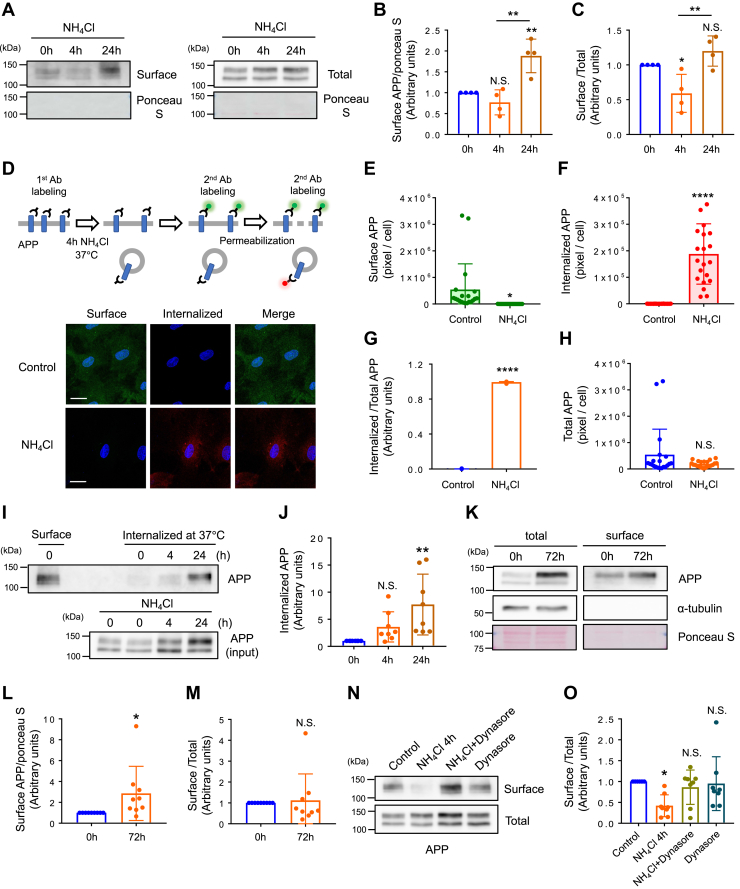
**Ammonia induces APP endocytosis *via* clathrin-dependent mechanism.***A*, representative blots of APP from cell surface biotinylation experiments after 4 h and 24 h NH_4_Cl treatments. Ponceau S was used as a loading control. *B*, quantification of surface APP. APP was normalized against ponceau S. n = 4, one-way ANOVA followed by Tukey's multiple comparisons test. ∗∗*p* < 0.01. N.S. = not significant. *C*, surface *versus* total mAPP ratio. n = 4, One-way ANOVA followed by Tukey's multiple comparisons test. ∗*p* < 0.05, ∗∗*p* < 0.01. N.S. = not significant. *D*, experimental designs are depicted in the *top panels*. Representative images of surface APP (*green*) and internalized APP (*red*) in control and NH_4_Cl-treated astrocytes. n = 9. The scale bars represent 20 μm. *E*, quantification of surface APP expression in control and NH_4_Cl-treated astrocytes. Control n = 20 cells, NH_4_Cl n = 21 cells. Unpaired *t* test, ∗*p* < 0.05. *F*, quantification of internalized APP in control and NH_4_Cl-treated astrocytes. Unpaired *t* test, ∗∗∗∗*p* < 0.0001. *G*, aligned dot plots showing internalized *versus* total APP ratio. Unpaired *t* test, ∗∗∗∗*p* < 0.0001. *H*, quantification of total APP in control and NH_4_Cl-treated astrocytes. Unpaired *t* test. N.S. = not significant. *I*, representative blot of internalized APP and surface APP (total) after 4 h and 24 h NH_4_Cl treatments. *J*, quantification of internalized APP. n = 8, One-way ANOVA followed by Tukey's multiple comparisons test. ∗∗*p* < 0.01, N.S. = not significant. *K*, representative blots of mature APP in control and 72 h NH_4_Cl-treated astrocytes determined by cell surface biotinylation assay. Alpha tubulin was used as a cytosolic marker. *L*, quantification of surface APP. APP was normalized against ponceau S. n = 9, paired *t* test, ∗*p* < 0.05. *M*, surface *versus* total mAPP ratio. n = 9, paired *t* test. N.S. = not significant. *N*, representative blots from cell surface biotinylation experiments determining the expression of mature APP in the 4 h NH_4_Cl- and Dynasore- (30 μM) treated astrocytes. *O*, surface *versus* total mAPP ratio. n = 8, One-way ANOVA followed by Tukey's multiple comparisons test, ∗*p* < 0.05. N.S. = not significant. APP, amyloid precursor protein; mAPP, mature APP.

APP is known as an integral membrane protein expressed in many cell types. Therefore, we examined whether NH_4_Cl alters the cell surface expression of astrocytic APP. Cell surface biotinylation revealed that a 24 h, but not 4 h, NH_4_Cl treatment significantly increased the expression of surface APP (Fig. 2, *A* and *B*). However, when surface APP was normalized to total APP, we found significantly reduced surface APP after 4 h of NH_4_Cl treatment (Fig. 2*C*). The internalization of surface APP at the 4 h timepoint was confirmed by an antibody feeding assay (Fig. 2*D*). A reduced amount of surface APP, as well as an increased amount of internalized APP, was detected after NH_4_Cl treatment (Fig. 2, *D*–*G*). The amount of total APP (surface + internalized) was not significantly altered after NH_4_Cl treatment (Fig. 2*H*). We performed further internalization assays and found increased APP internalization in 24 h treatment groups (Fig. 2,*I* and *J*). These results indicated that ammonia induces APP endocytosis, but surface APP is recovered upon prolonged NH_4_Cl treatment. To confirm our hypothesis, we analyzed surface APP after 72 h of NH_4_Cl treatment, a time point when ammonia significantly increased the expression of APP in astrocytes (Fig. 1*C*). We found that 72 h of ammonia treatment increased the amount of surface APP, but this was not significant when normalized to total mAPP (Fig. 2, *K*–*M*). In addition, we examined whether astrocytic APP was internalized *via* clathrin-dependent mechanisms. We treated astrocytes with dynasore, an inhibitor of dynamin GTPase activity, stimulated with NH_4_Cl for 4 h, and performed a steady state cell surface biotinylation assay. We found that dynasore inhibited the endocytosis of APP (Fig. 2, *N* and *O*). Additionally, we used chlorpromazine, a blocker of clathrin-mediated endocytosis and observed similar results (Fig. S3, *A* and *B*). Taken together, these data indicate that ammonia induces astrocytic APP endocytosis *via* clathrin-mediated mechanisms but that prolonged ammonia treatment recovers the expression of surface APP.

### Ammonia delays APP degradation but does not block proteolysis

**Figure 3:**
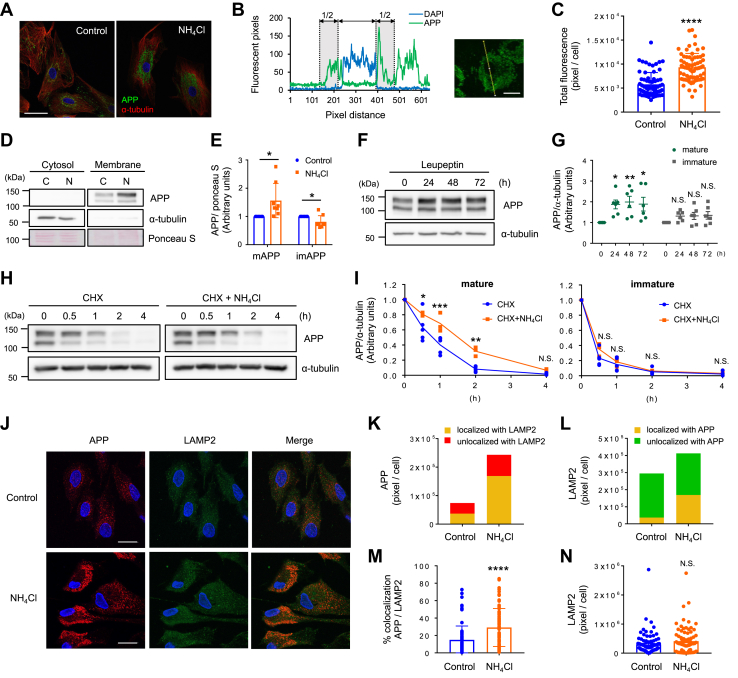
**Lysosomal APP degradation is delayed by ammonia.***A*, cellular localization of APP in control and 72 h NH_4_Cl-treated astrocytes. Images show APP (*green*), α-tubulin (*red*), and DAPI (*blue*). n = 6. The scale bar represents 50 μm. *B*, fluorescence profiles of APP over single astrocyte measured along the *yellow line*. Nucleus was identified by DAPI staining. The scale bar represents 20 μm. *C*, quantification of APP immunostaining in astrocytes with and without NH_4_Cl treatment. Control n = 67 cells, NH_4_Cl n = 68 cells. Unpaired *t* test, ∗∗∗∗*p* < 0.0001. *D*, subcellular fractionation of control and 72 h NH_4_Cl-treated astrocytes. Expression of APP and α-tubulin in cytosolic and membrane fractions are shown. *E*, quantification of mature and immature APP in membrane fraction. Expression of APP were normalized against ponceau S. n = 8, paired *t* test, ∗*p* < 0.05. *F*, representative Western blot images after lysosome inhibitor leupeptin (10 μM) treatments in cultured astrocytes. *G*, expression of APP were normalized against α-tubulin. Mature APP (*green*), immature APP (*gray*). n = 6, Two-way ANOVA followed by Sidak's multiple comparisons test, ∗*p* < 0.05, ∗∗<0.01. N.S. = not significant. *H*, turnover of APP with (*right*) or without (*left*) NH_4_Cl treatments. Protein synthesis was blocked by 5 μM cycloheximide (CHX). *I*, graphs quantify APP expression normalized against α-tubulin. n = 5, Two-way ANOVA followed by Sidak's multiple comparisons test, ∗*p* < 0.05, ∗∗*p* < 0.01, ∗∗∗ *p* < 0.001. *J*, cellular localization of APP (*red*) and LAMP2 (*green*) in astrocytes with and without 72 h NH_4_Cl treatment. Nuclei were counterstained with DAPI (*blue*). n = 6 (control), n = 5 (NH_4_Cl). The scale bars represent 20 μm. *K*, quantification of APP localized to the lysosome (*yellow*: localized with LAMP2) and not localized to the lysosome (*red*: unlocalized with LAMP2) in astrocytes. Control n = 91 cells, NH_4_Cl n = 77 cells. *L*, quantification of lysosome positive area localized to APP (*yellow*: localized with APP) and not localized to APP (*green*: unlocalized with APP). Control n = 91 cells, NH_4_Cl n = 77 cells. *M*, summary quantification of the percent of APP colocalizes with lysosome in the presence or absence of NH_4_Cl. Unpaired *t* test, ∗∗∗∗*p* < 0.0001. *N*, quantification of LAMP2-positive area in astrocytes with and without NH_4_Cl treatment. Unpaired *t* test. N.S. = not significant. APP, amyloid precursor protein.

Since ammonia promoted APP endocytosis, we planned to determine the site of APP accumulation in astrocytes. We first performed immunocytochemistry to observe cellular localization of APP after NH_4_Cl exposure. In control cultures, APP expression was broad, but cells exposed to NH_4_Cl showed distinct localization of APP around the nucleus (Fig. 3, *A*–*C*). We also performed subcellular fractionations and found that APP was not localized in the cytoplasmic fractions but accumulated in the membrane fractions after NH_4_Cl exposure (Fig. 3, *D* and *E*). Since a high concentration of NH_4_Cl is a lysosomotropic inhibitor, we examined whether 10 mM NH_4_Cl treatment blocked lysosomal protein degradation in the astrocytic cultures. We observed that after NH_4_Cl treatment, the lysosomal protease inhibitor leupeptin increased the expression of mAPP but not imAPP (Fig. 3, *F* and *G*). The potent proteasome inhibitor MG132 did not alter APP levels, suggesting that APP is degraded through autophagy-lysosome pathways (Fig. S4, *A* and *B*). We then performed a chase analysis of APP degradation by cycloheximide (CHX), a potent inhibitor of protein biosynthesis, and found that the half-life of APP is approximately 50 min for mAPP and 25 min for imAPP (Fig. 3,*H* and *I*). Upon NH_4_Cl exposure, the half-life of mAPP was extended to around 1.5 h, but degradation still occurred. These results suggested that NH_4_Cl exposure increased mAPP stability and delayed its proteolysis. By using the fluorescent dye LysoTracker, we found that 72 h NH_4_Cl-treated cultures increased LysoTracker-positive lysosomal area (Fig. S5, *A* and *B*). To determine whether APP is accumulated within these lysosomes, we performed coimmunostaining of APP with LAMP2, a lysosomal membrane protein. Increased APP accumulation in LAMP2-positive area was observed after NH_4_Cl exposure (Figs. 3, *J*–*N* and S5, *C* and *D*). Taken together, these data indicate that although NH_4_Cl delays the degradation of mAPP, APP can still be degraded in hyperammonemic conditions.

### Ammonia induces the translocation of internalized APP to the ER

**Figure 4:**
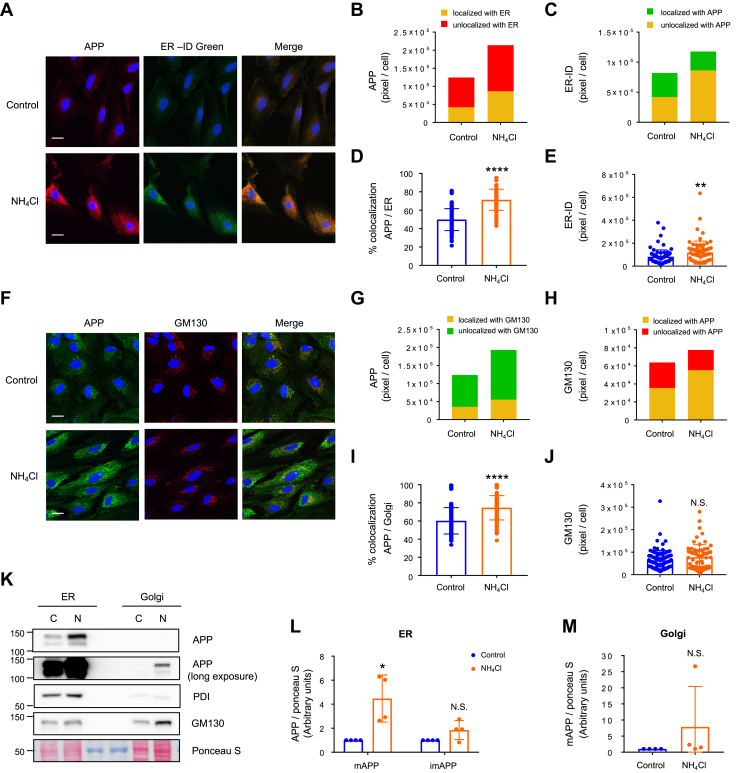
**Accumulation of APP in the astrocytic ER after NH**_**4**_**Cl treatment.***A*, cellular localization of APP (*red*) and ER-selective dye ER-ID Green (*green*) in the presence or absence of NH_4_Cl (72 h). Nuclei were counterstained with DAPI (*blue*). n = 4 (control), n = 5 (NH_4_Cl). The scale bars represent 20 μm. *B*, quantification of APP localized to the ER (*yellow*: localized with ER) and not localized to the ER (*red*: unlocalized with ER) in astrocytes with and without NH_4_Cl treatment. Control n = 90 cells, NH_4_Cl n = 67 cells. *C*, quantification of ER-ID positive area localized to APP (*yellow*: localized with APP) and not localized to APP (*green*: unlocalized with APP). Control n = 90 cells, NH_4_Cl n = 67 cells. *D*, summary quantification of the percent of ER colocalizes with APP in the presence or absence of NH_4_Cl. Unpaired *t* test, ∗∗∗∗*p* < 0.0001. *E*, quantification of ER-ID positive area in astrocytes with and without NH_4_Cl treatment. Unpaired *t* test, ∗∗*p* < 0.01. *F*, cellular localization of APP (*green*) and Golgi marker GM130 (*red*) in the presence or absence of NH_4_Cl (72 h). Nuclei were counterstained with DAPI (*blue*). n = 4 (control), n = 5 (NH_4_Cl). The scale bars represent 20 μm. *G*, fluorescence intensity quantification of APP localized to the Golgi (*yellow*: localized with GM130) and not localized to the Golgi (*green*: unlocalized with GM130) in astrocytes with and without NH_4_Cl treatment. Control n = 90 cells, NH_4_Cl n = 67 cells. *H*, quantification of GM130 localized to APP (*yellow*: localized with APP) and not localized to the APP (*red*: unlocalized with APP). *I*, summary quantification of the percent of APP colocalizes with the Golgi in the presence or absence of NH_4_Cl. Unpaired *t* test, ∗∗∗∗*p* < 0.0001. *J*, quantification of GM130 in astrocytes with and without NH_4_Cl treatment. N.S. = not significant. *K*, subcellular fractionation of control (C) and NH_4_Cl-treated (N) astrocytes. Expression of APP, ER (PDI), and *cis*-Golgi (GM130) in ER-enriched and Golgi-enriched fractions are shown. *L*, quantification of mature and immature APP in ER-enriched fraction. Expression of APP were normalized against ponceau S. n = 4, paired *t* test, ∗*p* < 0.05. N.S. = not significant. *M*, quantification of mature APP in Golgi-enriched fraction. Expression of APP were normalized against ponceau S. n = 4, paired *t* test. N.S. = not significant. APP, amyloid precursor protein; ER, endoplasmic reticulum.

Recent studies in neurons have revealed that Aβ can be produced not only at the plasma membrane of neurons but also in intracellular compartments such as the ER, Golgi apparatus, and the *trans*-Golgi network (TGN). The generation of Aβ42 in the ER is thought to contribute to the development of AD (28, 29, 30). Since APP was strongly expressed around the nucleus after NH_4_Cl treatment (Fig. 3*A*), we thought that the elevation of APP in this area might be caused by APP’s translocation to and accumulation in intracellular compartments. We found that the localization of APP to the ER was significantly increased after 72 h NH_4_Cl stimulation (Figs. 4, *A*–*D*, S6, *A* and *B*). An enlarged ER-positive area was also observed (Fig. 4*E*). In contrast, colocalization of APP with GM130, a marker for *cis*-Golgi, was less than the increase in colocalization with the ER (Figs. 4,*F*–*I*, S6, *C* and *D*). We did not observe an enlarged Golgi area after NH_4_Cl treatment (Fig. 4*J*). To determine whether the NH_4_Cl-induced enlargement of the ER-positive area was due to elevated ER stress, we examined the expression of ER stress markers, old astrocyte specifically induced substance (OASIS), and inositol-requiring enzyme 1 (IRE1). Both OASIS and IRE1 expression were not affected by NH_4_Cl treatment and the phosphorylation of IRE1, which represents its enzymatic activity, was also unaltered (Fig. S6, *E* and *H*). To further confirm the accumulation of APP in the ER following NH_4_Cl treatment, we performed subcellular fractionation and purified ER-enriched (PDI positive) and Golgi-enriched (GM130 positive) fractions. We observed that mAPP was highly expressed in the ER-enriched fraction and meager in the Golgi-enriched fraction, and NH_4_Cl stimulation significantly increased the expression of mAPP only in the ER-enriched fraction (Fig. 4, *K*–*M*).

**Figure 5:**
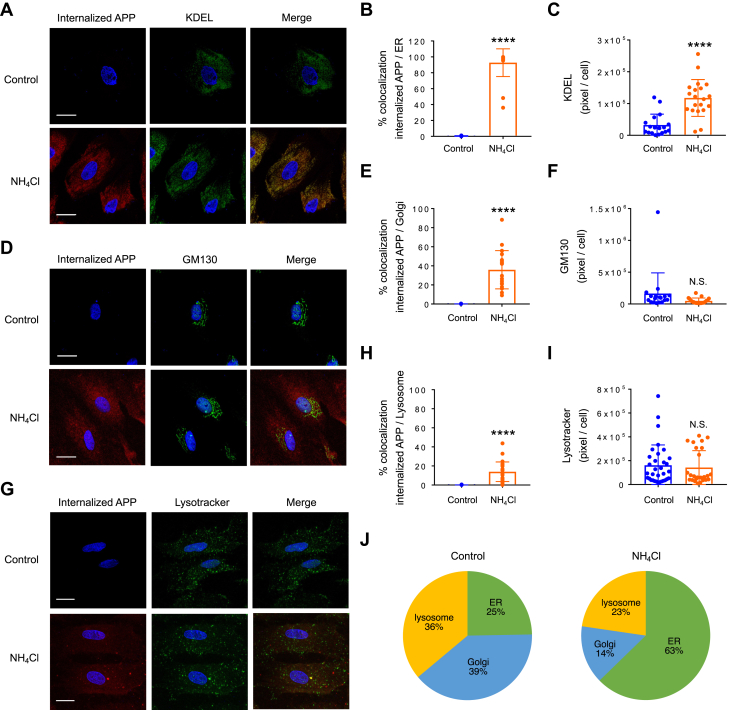
**Ammonia induces the translocation of APP from the plasma membrane to the ER.***A*, cellular localization of internalized APP (*red*) and ER marker KDEL (*green*) in the presence or absence of NH_4_Cl (4 h). Nuclei were counterstained with DAPI (*blue*). n = 15 (control), n = 10 (NH_4_Cl). The scale bars represent 20 μm. *B*, quantification of the percent of APP colocalizes with ER in the presence or absence of NH_4_Cl. Control n = 18 cells, NH_4_Cl n = 20 cells. Unpaired *t* test, ∗∗∗∗*p* < 0.0001. *C*, quantification of KDEL-positive area in astrocytes with and without NH_4_Cl treatment. Unpaired *t* test, ∗∗∗∗*p* < 0.0001. *D*, cellular localization of internalized APP (*red*) and Golgi marker GM130 (*green*) in the presence or absence of NH_4_Cl (4 h). Nuclei were counterstained with DAPI (*blue*). n = 9 (control), n = 13 (NH_4_Cl). The scale bars represent 20 μm. *E*, quantification of the percent of APP colocalizes with Golgi in the presence or absence of NH_4_Cl. Control n = 18 cells, NH_4_Cl n = 19 cells. Unpaired *t* test, ∗∗∗∗*p* < 0.0001. *F*, quantification of GM130-positive area in astrocytes with and without NH_4_Cl treatment. Unpaired *t* test. N.S. = not significant. *G*, cellular localization of internalized APP (*red*) and LysoTracker (*green*) in the presence or absence of NH_4_Cl (4 h). Nuclei were counterstained with DAPI (*blue*). n = 18 (control), n = 10 (NH_4_Cl). The scale bars represent 20 μm. *H*, quantification of the percent of APP colocalizes with lysosome in the presence or absence of NH_4_Cl. Control n = 33 cells, NH_4_Cl n = 26 cells. Unpaired *t* test, ∗∗∗∗*p* < 0.0001. *I*, quantification of LysoTracker–positive area in astrocytes with and without NH_4_Cl treatment. Unpaired *t* test. N.S. = not significant. *J*, expression ratio of internalized APP in ER *versus* Golgi *versus* lysosome in control and NH_4_Cl-treated astrocytes. APP, amyloid precursor protein; ER, endoplasmic reticulum.

To examine the translocation of APP from the plasma membrane to the ER, we performed an antibody-feeding assay and coimmunostained internalized APP with ER, Golgi, and lysosomal markers. Four hours of NH_4_Cl exposure induced a remarkable translocation of internalized APP to the ER (Fig. 5, *A* and *B*). Enlarged KDEL-positive areas were also identified (Fig. 5*C*). We also found APP localization in the Golgi and lysosomes, but the expression of internalized APP after NH_4_Cl treatment was less remarkable in these areas than that of the ER (Fig. 5, *D*–*H*). In addition, enlarged lysosome positive areas were not identified after 4 h of NH_4_Cl treatment (Fig. 5*I*). Interestingly, the localization ratio of internalized APP in the ER *versus* Golgi *versus* lysosome was equally distributed in the control condition; however, this ratio was shifted after 4 h of NH_4_Cl treatment and more than 60% of APP was localized in the ER (Fig. 5*J*). Together, these results show that ammonia induces internalized APP accumulation in the ER.

### Ammonia-induced astrocytic amyloidogenesis occurs in the ER

**Figure 6:**
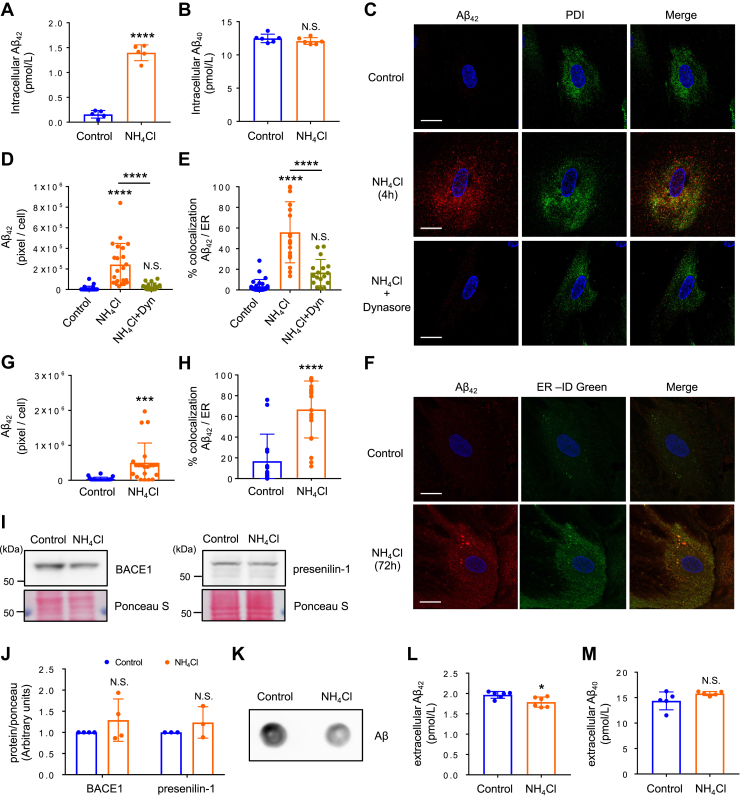
**Ammonia promotes the production of Aβ42 in the ER.***A*, quantitative ELISA analysis of intracellular Aβ42 expressed in control and 72 h NH_4_Cl-treated astrocytes. n = 5, unpaired *t* test, ∗∗∗∗*p* < 0.0001. *B*, quantitative ELISA analysis of intracellular Aβ40 expressed in control and NH_4_Cl-treated astrocytes. n = 5, unpaired *t* test. N.S. = not significant. *C*, representative images of cultured astrocytes expressing Aβ42 in the ER after 4 h NH_4_Cl treatment with and without 30 μM dynasore. Nuclei were counterstained with DAPI (*blue*). n = 3. The scale bars represent 20 μm. *D*, quantification of Aβ42-positive area. Control n = 27 cells, NH_4_Cl n = 22 cells, NH_4_Cl + Dynasore (Dyn) n = 17 cells. One-way ANOVA followed by Tukey's multiple comparisons test, ∗∗∗∗*p* < 0.0001. N.S. = not significant. *E*, summary quantification of the percent of Aβ42 colocalizes with ER in the presence or absence of NH_4_Cl. One-way ANOVA followed by Tukey's multiple comparisons test, ∗∗∗∗*p* < 0.0001. N.S. = not significant. *F*, representative images of cultured astrocytes expressing Aβ42 in the ER after 72 h NH_4_Cl treatment. Nuclei were counterstained with DAPI (*blue*). n = 4. The scale bars represent 20 μm. *G*, quantification of Aβ42-positive area. Control n = 20 cells, NH_4_Cl n = 21 cells. Unpaired *t* test, ∗∗∗*p* < 0.001. *H*, summary quantification of the percent of Aβ42 colocalizes with ER in the presence or absence of NH_4_Cl. Unpaired *t* test, ∗∗∗∗*p* < 0.0001. *I*, expression of BACE1 and presenilin-1 in ER-enriched fraction in control and NH_4_Cl-treated astrocytes. *J*, quantification of BACE1 (n = 4) and presenilin-1 (n = 3) in ER-enriched fractions. Expression of proteins were normalized against ponceau S. Paired *t* test. N.S. = not significant. *K*, representative dot blots of Aβ product in cultured medium. n = 3. *L*, quantitative ELISA analysis of extracellular Aβ42. n = 6, unpaired *t* test, ∗*p* < 0.05. *M*, quantitative ELISA analysis of extracellular Aβ40. n = 5, unpaired *t* test. N.S. = not significant. Aβ, amyloid beta; BACE1, beta-site APP cleaving enzyme 1; ER, endoplasmic reticulum.

We then examined the production of Aβ in astrocytes after 72 h of NH_4_Cl treatment. A selective increase in the production of Aβ42, the principal component of senile plaques in the brain of AD patients, was found in NH_4_Cl-treated astrocytes (Fig. 6, *A* and *B*). An increased Aβ42/Aβ40 ratio was also detected in these cells (control: 0.013 ± 0.0027, NH_4_Cl: 0.1173 ± 0.0065, n = 5, ∗∗∗∗*p* < 0.0001). We further examined whether Aβ42 accumulated in the astrocytic ER. We performed coimmunostaining of Aβ42 with ER markers and determined their colocalization after either 4 h or 72 h of NH_4_Cl stimulation (Figs. 6, *C*–*H* and S7*A*). We found that ammonia induces the production of Aβ42 in the ER and these amyloidogenic processes were inhibited by dynasore (Figs. 6, *C*–*E* and S7*A*). Furthermore, we examined if blockade of ER–Golgi trafficking by Brefeldin A modifies Aβ42 production in the ER. We found that Brefeldin A, which disassembles the Golgi complex (Fig. S7*B*), inhibited the production of Aβ42 in the ER (Fig. S7*A*). These data suggested that the retrograde transport of internalized APP from TGN to the ER may be the route of APP transportation. To confirm the presence of the two secretases that cleave APP to produce Aβ in the astrocytic ER, we purified ER-enriched fractions from cultured astrocytes and examined the expression of two secretases BACE1 (beta-site APP cleaving enzyme 1) and presenilin-1. We found that both proteins were located in the astrocytic ER and no change in their expression was detected after NH_4_Cl treatment (Fig. 6,*I* and *J*). These results indicate that ammonia induces the production and accumulation of Aβ42 in the astrocytic ER. Finally, we examined the amount of Aβ in the culture media. Interestingly, dot blot analysis revealed that NH_4_Cl reduced the release of Aβ from astrocytes (Fig. 6*K*). We also examined which of the two forms of Aβ are involved in this reduction and found that only the amount of Aβ42 was slightly reduced (Fig. 6, *L* and *M*). These data suggested that Aβ42 accumulates in astrocytes and it is not secreted.

### Hyperammonemia induces neurodegeneration and increases the expression of APP and Aβ in astrocytes

**Figure 7:**
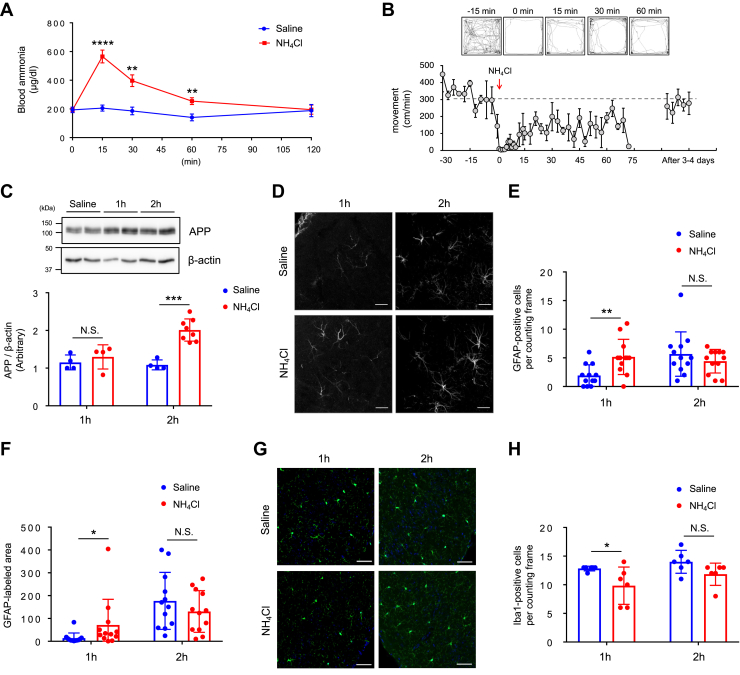
**Acute hyperammonemia induces neuroinflammation and astrocytic amyloidogenesis.***A*, time-dependent changes in the blood ammonia levels after systemic NH_4_Cl injection (5 mmol kg^−1^). Saline: n = 8, NH_4_Cl 15 min, 30 min, 60 min: n = 12, NH_4_Cl 120 min: n = 4, unpaired *t* test, ∗∗*p* < 0.01, ∗∗∗∗*p* < 0.0001. *B*, automated movement analysis in mice after systemic NH_4_Cl injection. Representative trace images from five time points are shown. n = 3. *C*, representative western blots and quantification of APP in the cortex 1 h and 2 h post NH_4_Cl injection. APP was normalized against β-actin. Saline n = 4, NH_4_Cl 1 h: n = 4, 2 h: n = 8, unpaired test, ∗∗∗*p* < 0.001; N.S., not significant. *D*, visualization of reactive astrocytes in the piriform cortex of 1 h- and 2 h-post saline or NH_4_Cl-injected mice by GFAP immunostaining. n = 3. The scale bars represent 50 μm. *E*, quantification of GFAP-positive cells per counting frame. n = 12, unpaired *t* test, ∗∗*p* < 0.01; N.S., not significant. *F*, quantification of GFAP-positive area per counting frame. n = 12, Mann-Whitney U test, ∗*p* < 0.05; N.S., not significant. *G*, visualization of microglia in the piriform cortex of 1 h- and 2 h-post saline or NH_4_Cl-injected mice by Iba1 immunostaining (*green*). All cells were counterstained for nuclei using DAPI (*blue*). n = 3. The scale bars represent 50 μm. *H*, quantification of Iba1-positive cells per counting frame. n = 6, Mann-Whitney U test, ∗*p* < 0.05; N.S. = not significant. APP, amyloid precursor protein; GFAP, glial fibrillary acidic protein.

To examine whether increased ammonia production is involved in the pathogenesis of AD *in vivo*, we intraperitoneally injected NH_4_Cl (5 mmol kg^−1^) in C57BL6/J mice, an acute model of hyperammonemia, and investigated its effect on APP. Shortly after the injection, a substantial increase in the level of blood ammonia was observed; these levels gradually returned to the baseline value in 2 h (Fig. 7*A*). Mice were video recorded after the injection, and their locomotion was scored, revealing a decrease in spontaneous movement immediately after NH_4_Cl injection, an effect which lasted for few minutes (Fig. 7*B*). Brains were harvested at 1 h- and 2 h-post NH_4_Cl challenge and showed significantly increased levels of APP in the cortex at the 2 h-post injection time point (Fig. 7*C*). The number and area of glial fibrillary acidic protein (GFAP)-positive reactive astrocytes was increased following 1 h NH_4_Cl injection (Fig. 7, *D*–*F*), while the number of Iba1-positive microglia was not modified (Fig. 7, *G* and *H*), suggesting that acute hyperammonemia induced by this agent leads to astrocytic neuroinflammation.

**Figure 8:**
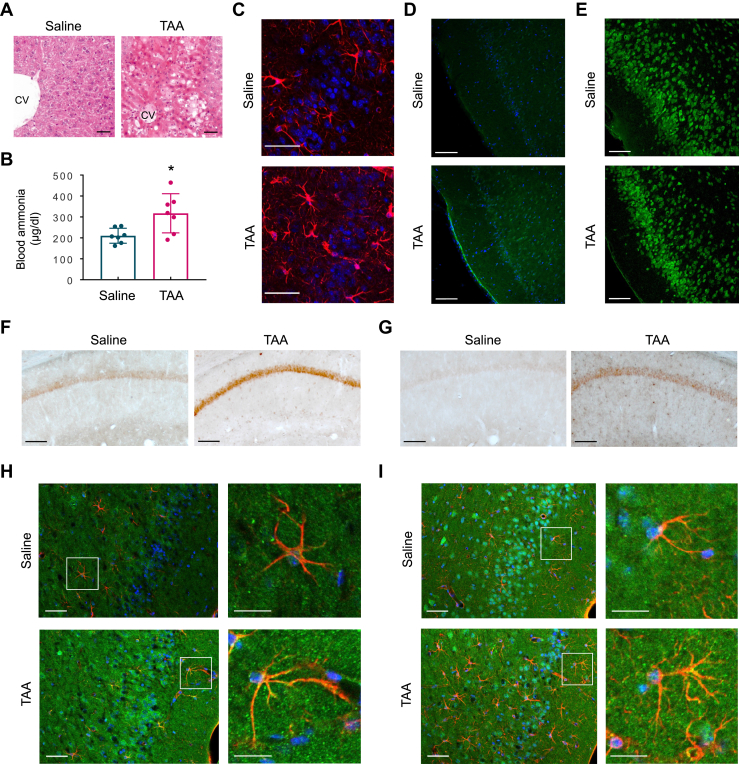
**Hyperammonemia induces neurodegeneration and astrocytic amyloidogenesis.***A*, representative H&E-stained sections of liver tissue in thioacetamide (TAA)-induced liver injury. Mice were sacrificed 24 h after the TAA (300 mg/kg, i.p.) or saline injection. CV: central vein. n = 3. The scale bars represent 50 μm. *B*, blood ammonia levels in saline and TAA mice. Saline n = 7, TAA n = 7, unpaired *t* test, ∗*p* < 0.05. *C*, visualization of reactive astrocytes in the piriform cortex of saline and TAA mice by GFAP immunostaining (*red*). All cells were counterstained for nuclei using DAPI (*blue*). n = 5. The scale bars represent 50 μm. *D*, Fluoro-Jade C staining in the piriform cortex of saline and TAA mice. All cells were counterstained for nuclei using DAPI (*blue*). n = 3. The scale bars represent 100 μm. *E*, NeuN immunostaining in the piriform cortex of saline and TAA mice. The scale bars represent 100 μm. n = 4. *F* and *G*, representative immunohistochemical images of APP (F) and Aβ (G) in the hippocampal CA1 sections of saline and TAA mice. n = 3. The scale bars represent 50 μm. *H*, expression of APP in the piriform cortex of saline and TAA mice. Images showed GFAP (*red*), APP (*green*), and DAPI (*blue*). n = 5. The scale bars represent 50 μm (high magnification images: 20 μm). *I*, expression of Aβ in the piriform cortex of saline and TAA mice. Images showed GFAP (*red*), Aβ (*green*), and DAPI (*blue*). n = 5. The scale bars represent 50 μm (high magnification images: 20 μm). Aβ, amyloid beta; APP, amyloid precursor protein; GFAP, glial fibrillary acidic protein.

In order to further verify whether hyperammonemia induces the pathology of AD, we used the thioacetamide (TAA)-induced hepatic encephalopathy model in C57BL6/J mice (31). Compared to saline-injected controls, TAA administration caused severe liver damage, including acute focal necrosis, vacuolization in some hepatocytes with mild inflammatory cell infiltration (Fig. 8*A*), and a marked increase in the concentration of blood ammonia (Fig. 8*B*). To investigate whether hyperammonemia leads to neuroinflammation and neurodegeneration, we labeled reactive astrocytes with GFAP and degenerating neurons using Fluoro-Jade C staining. We identified an increased number of reactive astrocytes, characterized by thickening of the cell body and cellular processes, as well as a progression of neurodegeneration in TAA groups (Figs. 8, *C* and *D* and S8*A*). The extent of neuroinflammation was further determined by the reduced expression of NeuN, a neuron-specific nuclear protein, in TAA-treated mouse brains (Figs. 8*E* and S8*B*).

We then examined if the expression of proteins related to AD pathology were increased in TAA-treated mice. The expression of both APP and Aβ were elevated compared to saline injection group (Fig. 8, *F* and *G*). To determine the colocalization of these proteins with astrocytes, the only non-neuronal brain cells capable of detoxifying ammonia, we employed immunostaining. We found a strong signal for both APP and Aβ in TAA-injected mouse astrocytes (Figs. 8,*H* and *I*, S8, *C* and *D*). Together, these data suggest that hyperammonemia triggers neuroinflammation and neurodegeneration, as well as elevation of APP and Aβ levels in astrocytes.

## Discussion

Many studies have indicated that ammonia could be a pathogenic factor in the etiology of AD (1, 2, 32). However, the mechanisms by which ammonia induces the production of AD-related molecules is ill defined. In this study, we demonstrated for the first time that ammonia triggers the endocytosis of astrocytic APP and promotes the translocation of internalized APP to the ER. Aβ42 also accumulated in the astrocytic ER upon exposure to ammonia, suggesting that mistargeted APP is the source of Aβ42 in this compartment. All the changes observed in astrocytes in this study may be potential mechanisms by which ammonia leads to components of AD pathogenesis.

We found that ammonia enhances the expression of astrocytic APP *in vivo* and biochemical analysis determined that this elevation was specific to mAPP, but not imAPP. The level of APP mRNA was unaltered by ammonia. From these results, we hypothesized that the process of APP maturation and the trafficking of imAPP from ER to Golgi apparatus is unaffected by ammonia. The neuronal mAPP secreted from the Golgi apparatus can be targeted to the plasma membrane, the endosome, or the lysosomes (28). It has been previously reported that only 10% of APP goes to the plasma membrane, with the majority remaining in the Golgi apparatus and/or TGN (33). Studies performed in nonpolarized cells suggest that APP is internalized and reaches the endosome due to the presence of a YENPTY internalization motif near the C-terminus (34, 35). After endocytosis, APP can return to the cell surface, be degraded in the lysosome, or be transported to the TGN. Ammonia has been shown to affect phagocytotic and pinocytotic activities in astroglioma cell lines (36). By using surface biotinylation and antibody-feeding assays, we found that ammonia induces the internalization of astrocytic APP. The endocytosis of astrocytic APP was through the clathrin-mediated pathway, as the dynamin inhibitor dynasore and the clathrin-mediated endocytosis inhibitor chlorpromazine recovered surface mAPP levels that were reduced by ammonia. Interestingly, even though the amount of internalized APP was increased after prolonged ammonia exposure, the expression of APP on the plasma membrane was also increased by ammonia. These incompatible phenomena could result from increased recycling of APP or from increased insertion of newly synthesized mAPP occurring during ammonia stimulation. Since the ratio of surface *versus* total APP was not altered after prolonged ammonia treatment, the proportion of APP that is transported to the plasma membrane may be well defined based on the amount of total APP expressed in astrocytes. In addition, recent studies have revealed the role of astrocytic APP in calcium signaling (37). APP has also been proposed to act as a cell adhesion molecule; cell adhesion molecules are involved in neuronal development, including migration, neurite growth, growth cone pathfinding, and synaptogenesis (38). Although the physiological role of astrocytic APP is not well understood, the maintenance of surface APP expression levels after prolonged NH_4_Cl treatment suggests that APP plays an important role in astrocyte function.

Several ammonia transporters including NKCC1 and AQP4 have been reported in astrocytes. NKCC1 is known to be involved in cell swelling in several neurological disorders and ammonia is thought to be an NKCC1 activator (39). Furthermore, NKCC1 has been shown to transport NH_4_^+^ in astrocytes but not in neurons (26). AQP4 is a water channel that is widely distributed in cells at the blood–brain and brain–cerebrospinal fluid interfaces where water movement occurs (40). There are 13 mammalian aquaporins (AQP0-AQP12) and AQP1, AQP3, AQP6, AQP7, AQP8, and AQP9 have been reported to be permeable to NH_3_, although the NH_3_ permeability of AQP1 has been questioned (41, 42). Among these aquaporins, AQP1, AQP4, and AQP9 have been found in astrocytes. In the human CNS, AQP4 is expressed in both physiological and pathological conditions, while astrocytic expression of AQP1 and AQP9 is mainly associated with a pathological state (43). By using the NKCC1 inhibitor bumetanide and the AQP4 inhibitor TGN-020, we found that these two ammonia transporters are not involved in the upregulation of mAPP. In the present study, we did not test AQP1 and AQP9 inhibitors, due to the expression profiles of these two aquaporins. However, since bumetanide and TGN-020 did not inhibit the upregulation of mAPP after NH_4_Cl exposure, we believe that extracellular ammonia-mediated signaling may contribute to mAPP accumulation in astrocytes. Supporting this hypothesis, ammonia has been found to act through dopamine D3 receptors (44). Ammonia has also been shown to transactivate the EGF receptor *via* Na, K-ATPase/Ouabain signaling (45). Clearly, further mechanistic studies are required to determine how ammonia specifically modify astrocytic APP trafficking and accumulation in the ER.

Most of APP is known to undergo nonamyloidogenic processing *via* consecutive cleavages by α- and γ-secretases, resulting in nonpathogenic fragments. However, APP also undergoes sequential proteolytic cleavage by β- or γ-secretases, a process which generates neurotoxic Aβ peptides (46). Cell culture experiments have shown that APP retrieved from the cell surface *via* clathrin-mediated endocytosis is cleaved by β- and γ-secretase within late and early endosomes to produce Aβ (47, 48). Aβ generated in the endocytic pathway is then brought to the cell surface, where it is released into the extracellular fluid (49). We found that the amount of Aβ in culture medium was reduced upon NH_4_Cl exposure, indicating that the site of Aβ production in astrocytes is not within endosomes but occurs within other intracellular compartments such as ER and Golgi apparatus. Since the plasma membrane has been demonstrated to be the predominant site for nonamyloidogenic processing of APP by α-secretase (50), it is possible that the production of nonamyloidogenic peptides occur within APP located at the plasma membrane during NH_4_Cl treatments.

Several studies have demonstrated that Aβ42 is generated in the ER, whereas Aβ40 is produced in the TGN (29). These amyloidogenic mechanisms have been proposed to be unique to neurons (29). In addition, neuroblastoma cells doubly transfected with human APP and WT presenilin-1 have been found to generate Aβx-42, a truncated insoluble Aβ42, in the ER (51). Interestingly, these insoluble Aβ42 were not secreted (51). In our work, we found that after NH_4_Cl treatment, astrocytic APP is preferentially accumulated in the ER and enhanced Aβ production in astrocytes is specific to Aβ42. Brefeldin A treatment in the NH_4_Cl-treated astrocytes blocked Aβ42 generation in the ER, suggesting that mAPP are transported from TGN to the ER *via* Rab2- or Rab6-linked retrograde vesicles (52). Indeed, we found small increase in internalized APP localization with GM130. Although it is unclear why the ER is the predominant location for Aβ generation after ammonia treatment, this process would limit its accumulation if lysosomes thereby preventing its degradation. Furthermore, ammonia exposure did not change the production of Aβ40. Though we found a slight increase in the amount of APP targeted to the Golgi apparatus, we did not observe changes in the size of the Golgi. Therefore, it appears that ammonia does not accelerate the production of Aβ in the TGN. Nevertheless, our results provide novel evidence that non-neuronal cells such as astrocytes are capable of producing Aβ in their intracellular compartments, depending on changes in the cellular environment such as hyperammonemia.

Additionally, we found that ammonia treatments suppress the mitotic activity of astrocytes. The number of dead cells was also increased by ammonia. These toxic effects only appeared after prolonged exposure. Therefore, the earlier accumulation of Aβ42 in the astrocytic ER may induce apoptosis, resulting in increased dead cells after 48 h of NH_4_Cl treatment.

In addition to the accumulation of internalized APP and Aβ42 within the ER, we observed an enlarged ER after ammonia treatment. The ER is the primary subcellular organelle responsible for protein folding, biosynthesis of lipids and sterols, and calcium storage (53). Altered ER function leads to the accumulation of unfolded or misfolded proteins in the ER lumen, a phenomenon referred to as ER stress. Upon ER stress, the lumen of the ER is remarkably enlarged. We found that, after ammonia exposure, the activity and expression of ER stress transducer IRE1 is not increased and the expression of OASIS, an astrocyte and osteoblast specific ER stress transducer, is not altered either. Therefore, the enlarged ER induced by ammonia is not the result of ER stress. Since the accumulated APP in the ER is mAPP and neither an unfolded nor misfolded forms, other signal transduction cascades may be activated in these cells after exposure to ammonia.

The secretase-independent degradation of APP has been thought to prevent the formation of cytotoxic peptide fragments. For instance, ubiquitin-1, a ubiquitin-like protein, has been shown to delay APP maturation and proteasomal degradation by stimulating APP lysine 63-linked polyubiquitination (54, 55). Previous studies in chinese hamster ovary cells show that APP is rapidly degraded by the ubiquitin-proteasome system in response to ER stress (56). In addition, abnormalities of the endolysosomal and autophagy system are reported in AD (57), and APP processing and Aβ production are found to be regulated by the endolysosomal system (58). In the present study, we found that astrocytic APP is degraded through the lysosomal-autophagy pathway and this degradation is delayed by ammonia. Accumulated APP was observed in LAMP2-positive lysosomes. However, when we chased APP using an antibody-feeding assay, the ammonia-induced internalized APP were mainly localized in the ER. These data suggest that prolonged ammonia treatment alters lysosomal function and induces APP accumulation in lysosomes; however, most of this APP is likely to be newly synthesized APP and not internalized APP.

Collectively, this study provides evidence for an astrocyte-specific process that leads to the production of Aβ42 and direct evidence that ammonia induces the pathogenesis of AD by regulating astrocyte function.

## Experimental procedures

### Animals

All experiments were carried out in accordance with the Guidelines for the Care and Use of Laboratory Animals of Niigata University. Animal care and experimental protocols were approved by the Animal Experiment Committee of the Niigata University (approval No. SA00688, SA00820). Animals used in this study were 10-week-old male C57BL6/J mice. In TAA-induced liver injury, TAA (Sigma-Aldrich) was injected intraperitoneally at 300 mg/kg body weight. To induce acute hyperammonemia in mice, 5 mmol/kg of ammonium chloride (Sigma-Aldrich) was injected intraperitoneally. Blood samples were collected from the tail vein and blood ammonia content was measured by DRI-CHEM NX10N (FUJIFILM) according to the manufacturer’s instructions. At the end of the experiments, mice were anesthetized with isoflurane and tissues were harvested for analyses. Liver damage was measured by H&E staining (FUJIFILM Wako Pure Chemical Corporation).

### Astrocyte-enriched cortical glial cultures

Cerebral cortical astrocytes were prepared from E18 to 19 Sprague-Dawley (SD/Jcl, CLEA Japan) rats. Dissected cortex was treated with 0.25% trypsin (Gibco), triturated in minimum essential medium (MEM, Sigma-Aldrich) containing 10% fetal bovine serum (Cytiva HyClone), and transferred into flasks (Thermo Fisher). Cell cultures were grown to confluence at 37 °C in a humidified 5% CO_2_ atmosphere. After 7 to 10 days, flasks were washed with cold Hank’s balanced salt solution (Gibco) and fed with cold MEM before shaking at 115 rpm for 2 days. Remaining adherent cells were dissociated using 0.025% trypsin-EDTA (Gibco) and plated onto coverslips or culture dishes. Cells were used after 4 to 10 days in culture unless specifically stated. For the cellular treatments, following chemicals were used: NH_4_Cl (Sigma-Aldrich), ammonium acetate (Sigma-Aldrich), bumetanide (Sigma-Aldrich), TGN-020 (Sigma-Aldrich), L-methionine sulfoximine (Sigma-Aldrich), glutamine (FUJIFILM Wako Pure Chemical Corporation), and sodium hydroxide (FUJIFILM Wako Pure Chemical Corporation).

### Animal behavior

The open field test was performed to determine basal activity in hyperammonemic mice. Mice were placed at the corner of open-field chamber, which consisted of a square platform with 50 cm (width) × 40 cm (height) walls illuminated at a light intensity of 5 lux (O’hara & Co), and left free to explore for 30 min before 5 mmol/kg NH_4_Cl intraperitoneal injection and then 75 min after the injection. Total distance traveled was recorded and calculated automatically using Image OFCR software (O’Hara & Co). Each movement distance (cm)/min was averaged in 2 to 3 min bins, except for the period of 10 min after NH_4_Cl injection. The chamber was cleaned using sodium hypochlorite solution between each session.

### Immunohistochemistry

Under deep inhalation of sevoflurane, mice were transcardially perfused with 4% paraformaldehyde (PFA) in 0.1 M phosphate buffer (pH 7.2). Cryosections were prepared at 35 μm using a cryostat (Microm HM500; Thermo Fisher Scientific). For immunostaining, sections were permeabilized with 0.3% Triton X-100 (Sigma-Aldrich) in PBS (Sigma-Aldrich) for 15 min and then blocked with 0.5% skim milk (Megmilk Snow Brand) for another 15 min at room temperature. The sections were incubated with the following primary antibodies in 0.1% Triton X-100 containing PBS for overnight at 4 °C: monoclonal mouse anti-GFAP (Millipore, MAB360), polyclonal rabbit anti-GFAP (Millipore, AB5804), anti β-Amyloid (Santa Cruz Biotechnology, SC28365), anti-APP (Thermo Fisher Scientific, Rb-9023-P0), anti-APP (abcam, Y188), anti-Iba1 (FUJIFILM Wako, 019-19741), and anti-NeuN (Millipore, MAB377). Sections were then incubated with a mixture of Alexa Fluor 488- and Alexa Fluor 594-labeled species-specific secondary antibodies (Thermo Fisher Scientific) for 2 h. Images were taken with a confocal laser-scanning microscope (Zeiss LSM710; Carl Zeiss).

### Fluoro-Jade C staining

Fluoro-Jade C staining was performed according to the manufacturer’s protocol (Fluoro-Jade C staining Kit, Biosensis). Briefly, sections attached to MAS-coated glass slides (Matsunami glass) were immersed in 1% NaOH/80% ethanol for 5 min, and sequentially rinsed for 2 min with 70% ethanol and 2 min with distilled water, and then incubated in 0.06% potassium permanganate/distilled water for 10 min. After rinsing with water for 2 min, sections were incubated in 0.0001% FJC/0.1% acetic acid for 10 min. Finally, the sections were rinsed for 1 min with distilled water three times, dried at 55 °C for 5 min, cleared in Xylene (FUJIFILM Wako) for 1 min, and mounted with DPX, a nonaqueous mounting medium (Merck, 100579). All reactions and incubations were performed at room temperature in the dark.

### Western blotting

Standard Western blot protocol was used as described previously (59). Protein samples were subjected to SDS-PAGE and transferred to supported nitrocellulose membranes (GE Healthcare Life Sciences). Membranes were stained with ponceau S (Sigma-Aldrich) for protein detection, then blocked with blocking buffer (5% bovine serum albumin in Tris Buffered Saline with Tween 20) and probed with primary antibodies against APP (abcam, Y188), β-actin (Sigma-Aldrich, Clone AC-15), α-tubulin (Sigma-Aldrich, T5168), GM130 (BD Biosciences, Clone 35), PDI (Cell Signaling, C81H6), IRE1 (Novus biological, NB100-2324), phosphor-IRE1 (Novus biological, NB1002323), OASIS (Santa Cruz Biotechnology, sc-514635), presenilin-1 (Santa Cruz Biotechnology, sc-365450), BACE1 (Santa Cruz Biotechnology, sc-33711). Membranes were then probed with horseradish peroxidase–conjugated secondary antibodies (GE Healthcare) and visualized by ECL (SuperSignal West Dura Extended Duration Substrate, Thermo Fisher Scientific). Blots were quantified using the CCD-based Amersham Imager 680 system (GE Healthcare Life Sciences) and the intensity of bands was measured using Image J.

### Preparation of ER-enriched and Golgi-enriched fraction

ER-enriched and Golgi-enriched fractions were prepared by modifying the use of the Endoplasmic Reticulum Isolation Kit (Sigma Aldrich, ER0100). First, the post-mitochondrial fraction was collected according to the manufacture’s protocol. The post-mitochondrial fraction was then centrifuged for 60 min at 100,000*g* in an ultracentrifuge at 4 °C. The pellet was then resuspended in lysis buffer (10 mM Tris–HCl, pH 8.0, 150 mM NaCl, 1% Triton X-100, 5 mM EDTA, 10 mM NaF, 2 mM Na_3_VO_4_, 10 mM Na_4_P_2_O_7_), including four kinds of protease inhibitors (antipain, leupeptin, pepstatin A, PMSF), and kept as an ER-enriched fraction. The remaining supernatant was stored as a Golgi-enriched fraction.

### Steady-state cell surface biotinylation assay and internalization assay

Labeling of surface proteins for steady-state cell surface biotinylation were performed as reported previously in cultured cortical neurons (60). Briefly, biotinylated proteins were precipitated with Pierce NeutrAvidin UltraLink Resin (Thermo Fisher Scientific), and the samples were separated by SDS-PAGE. Surface and total proteins were visualized by Western blotting. To block clathrin-mediated endocytosis, dynasore (Adipogen Life Sciences) and chlorpromazine hydrochloride (Tokyo chemical industry) were used. For the internalization assay, surface proteins were labeled with EZ-Link Sulfo-NHS-SS-Biotin (Thermo Fisher Scientific) for 20 min at 4 °C. Excess biotin was quenched with 25 mM Glycine, then incubated with NH_4_Cl at 37 °C for appropriate times. For the 0 min time point, cells were kept at 4 °C as a control. After incubation, cells were quickly washed with ice-cold PBS to stop internalization, and remaining cell surface biotin was cleaved with 50 mM glutathione (Sigma Aldrich) for 30 min at 4 °C. Cells were then extracted in lysis buffer, as described previously (59, 61). Biotinylated proteins were precipitated with Pierce NeutrAvidin UltraLink Resin, and samples were separated by SDS-PAGE. To detect total surface proteins, we prepared cells labeled with EZ-Link Sulfo-NHS-SS-Biotin without cleavage.

### Protein stability assay

For CHX chase analysis, cultured astrocytes treated with or without NH_4_Cl (10 mM) were incubated with 5 μM CHX at the indicated time points. Cell lysates were prepared, and the expression of APP was analyzed by Western blotting. For loading control, α-tubulin (Sigma-Aldrich) was used. To evaluate the effect of the ubiquitin proteasome pathway and the autophagy-lysosome pathway on APP degradation, 0.5 μM MG132 (Sigma-Aldrich) and 10 μM leupeptin (Sigma-Aldrich) were applied in the cell culture medium. The expression of APP was analyzed by Western blotting.

### Preparation of astrocytic membrane and cytosolic fractions

Astrocytes were washed with ice-cold PBS (Gibco), resuspended with homogenization buffer (0.32 M sucrose, 10 mM Hepes, 2 mM EDTA), and homogenized with a Teflon homogenizer (5 strokes). Homogenates were then spun at 900 rpm for 10 min to remove nuclei, and the collected supernatants were centrifuged at 50,000 rpm for 30 min using an Optima MAX-E Ultracentrifuge (Beckman Coulter). Membrane pellets were resuspended in lysis buffer as described previously (61, 62) and subjected to Western blotting.

### Brightfield microscopy

#### Cell counting

Astrocytes grown in 6 cm dishes were harvested by trypsin-EDTA application and centrifuged at 1000 rpm for 5 min. Cell pellets were resuspended in 1 ml of Hank’s balanced salt solution and a portion of cell suspension (20 μl) was counted using a TC20 automated cell counter (Bio-Rad Laboratories).

### Scratch assay

Astrocytes were plated in 6 cm dishes and grown in the incubator at 37 °C in a humidified 5% CO_2_ atmosphere until they reached ∼90% confluence. A straight-line scratch was made on a confluent monolayer of cells using a 200 μl sterile disposable pipette. Cells were then washed with 1 ml MEM to remove debris. Photos were taken using an ECLIPSE Ts2 (Nikon) with Moticam 1080 (Shimadzu-rika) at 0, 24, and 48 h after NH_4_Cl treatments.

### RT-PCR

Total RNA from cultured astrocytes was extracted using an RNeasy mini kit (Qiagen). RNA was quantified using a NanoDrop One (Thermo Fisher Scientific) and retro-transcribed using oligo (dT)_20_ Primer (Thermo Fisher Scientific), M-MLV Reverse Transcriptase (Promega), Recombinant ribonuclease inhibitor (Invitrogen), and dNTPs (TOYOBO). The cDNA was then subjected to PCR with Taq (Takara), dNTPs (TOYOBO), and primers: for *APP*, 5′-GGATGCGGAGTTCGGACATG-3′ and 5′-GAAACTCGTCTCAGTCTTG-3′ and for *GAPDH*, 5′-GGCAAGTTCAATGGCACAGT-3′ and 5′CTCAGATGACCGCAGAAGTGGT-3’. PCR products were separated by electrophoresis on an agarose gel and stained with GelRed Nucleic Acid Stain (Biotium) for visualization. The intensity of bands was measured using Image J. The expression level of APP mRNA was normalized to the level of GAPDH.

### Immunocytochemistry

Astrocytes on coverslips were fixed in 4% PFA for 15 min at room temperature. Fixed cells were permeabilized in 0.2% Triton X-100 or 0.01% Saponin (Sigma-Aldrich) for 30 min at room temperature. Following blocking in PBS supplemented with 0.2% Triton X-100 and 1% bovine serum albumin, cells were incubated with primary antibodies overnight at 4 °C. Cells were then incubated with fluorescently tagged secondary antibodies (VECTOR Laboratories) for 1 h at room temperature. All cells were counterstained with DAPI. Confocal images were taken using a laser-scanning confocal microscope (Zeiss LSM700) and images were analyzed using Zen-Black imaging software or Image J. Colocalization was also measured using the Image J Fiji’s “Coloc2” plugin to quantify Mander’s overlap coefficient. For the antibody-feeding assay, APP on the plasma membrane was labeled with anti-APP (Thermo Fisher Scientific, RB-9023-P0), which detects the N-terminal region of APP.

### Labeling of ER

Astrocytes on coverslips were fixed with 4% PFA for 15 min at room temperature and subjected to membrane permeabilization with 0.2% Triton X-100 for 30 min. Cells were then stained with the ER-ID Green Assay Kit (Enzo Life Sciences) for 1 h, washed with PBS, and counterstained with DAPI. Confocal images were taken using a laser-scanning confocal microscope (Zeiss LSM700) and images were analyzed using Zen-Black imaging software or Image J. In addition to ER-ID staining, immunostaining for calnexin was performed (see immunocytochemistry section).

### Labeling of lysosome with Lysotracker

Astrocytes on coverslips were stained with LysoTracker Red DND-99 (Invitrogen). Culture medium was supplemented with 200 nM of probes for 1 h at 37 °C in a humidified incubator with 5% CO_2_. Stained cells were washed with PBS and fixed with 4% PFA for 15 min at room temperature. Cells were washed with PBS and counterstained with DAPI. Confocal images were taken using a laser-scanning confocal microscope (Zeiss LSM700) and images were analyzed using Zen-Black imaging software or Image J.

### Quantification of Aβ by ELISA

Cultured medium was collected and centrifuged at 1400 rpm for 1 min to remove dead cells. Astrocytes on culture dishes were washed twice with ice-cold PBS and homogenized with Buffer A (20 mM Tris–HCl pH 8.0, 150 mM NaCl, 5 mM EDTA, 0.1% SDS), including four kinds of protease inhibitors (antipain, leupeptin, pepstatin A, PMSF). Cells were then centrifuged at 13,000 rpm for 10 min and supernatant was used for Aβ measurement. The amount of Aβ40 and Aβ42 in culture medium and cell lysates was measured by sandwich ELISA (FUJIFILM Wako), according to the manufacturer’s instructions. Each sample including standards were tested in duplicate and the average values were used.

### Statistics

Data were subjected to unpaired *t* test, Mann-Whitney U test, one-way or two-way ANOVA followed by Dunnett's multiple comparisons test (one-way), Tukey's multiple comparisons test (one-way) or Sidak’s multiple comparison tests (two-way), as appropriate with *p* < 0.05 as statistically significant. All statistical analyses were performed using GraphPad Prism 7.0 software. Values on the graph represent the mean ± SD. All experiments were conducted a minimum of three times using different batches of cultures and animals. Data normality was assessed using the D’Agostino- Pearson and the Shapiro-Wilk normality tests.

## Data availability

All data generated or analyzed during this study are included in this article and its supplementary information files.

## Supporting information

This article contains supporting information.

## Conflict of interest

The authors declare no conflicts of interest associated with this article.
