# Supplementary material for: Ammonia induces amyloidogenesis in astrocytes by promoting amyloid precursor protein translocation into the endoplasmic reticulum
Source: J Biol Chem. 2022 Apr 12;298(5):101933. doi: 10.1016/j.jbc.2022.101933 (PMC9117890; doi:10.1016/j.jbc.2022.101933)
Supplement: Supporting information [file mmc1.docx]

**Supporting information**

**Figure legends**

**Figure S1. Characterization of cultured cortical astrocytes obtained from rat E18-19 embryos.**

*A*, Immunostaining of purified cultured cortical astrocytes with the astrocyte marker GFAP (green) and the microglial marker CD11b (red). Nuclei were counterstained with DAPI (blue). n = 5. Scale bars = 50 μm. *B*, Immunostaining of prolonged-cultured cortical astrocytes (4 weeks) with GFAP (green) and CD11b (red). Nuclei were counterstained with DAPI (blue). n = 3. Scale bars = 50 μm. *C*, Immunostaining of a mixed glia with GFAP (green) and CD11b (red). Nuclei were counterstained with DAPI (blue). n = 3. Scale bars = 50 μm. *D*, Immunostaining of a mixed glia with GFAP (green) and the oligodendrocyte marker O4 (red). Nuclei were counterstained with DAPI (blue). n = 3. Scale bars = 50 μm. *E*, Immunostaining of purified cultured cortical astrocytes with GFAP (green) and O4 (red). Nuclei were counterstained with DAPI (blue). n = 3. Scale bars = 50 μm.

**Figure S2. The effect of pH, glutamine, and glutamine synthetase blocker L-Methionine sulfoximine in APP expression.**

*A*, Representative western blots of APP and α-tubulin after time-dependent alkaline medium (pH8.7) treatments in cultured astrocytes. *B*, Quantification of mature APP (green) and immature APP (gray) pooled from three experiments. Two-way ANOVA followed by Sidak’s multiple comparisons test. N.S. = not significant. *C*, Representative western blots of APP and α-tubulin after 4h alkaline medium (pH8.7) treatment in cultured astrocytes. *D*, Quantification of mature APP (green) and immature APP (gray) pooled from four experiments. Unpaired *t* test. N.S. = not significant. *E*, Representative western blots of APP and α-tubulin in cultured astrocytes that are starved for 36h and stimulated with 4 mM glutamine for various time points. *F*, Quantification of mature APP (green) and immature APP (gray) pooled from four experiments. Two-way ANOVA followed by Sidak’s multiple comparisons test. N.S. = not significant. *G*, Representative western blots of APP and α-tubulin after time-dependent 3 μM L-Methionine sulfoximine (MSO) treatments in cultured astrocytes. *H*, Quantification of mature APP (green) and immature APP (gray) pooled from four experiments. Two-way ANOVA followed by Sidak’s multiple comparisons test. N.S. = not significant.

**Figure S3. The effect of chlorpromazine on NH_4_Cl-induced APP endocytosis**

*A*, Representative blots from cell surface biotinylation experiments determining the expression of mature APP in the 4h NH_4_Cl and Chlorpromazine (CPZ; 10 μg/ml) treated astrocytes. *B*, Surface versus total mAPP ratio. n = 4, One-way ANOVA followed by Tukey's multiple comparisons test, **p* < 0.05. N.S. = not significant.

**Figure S4. The effect of proteasome inhibitor on APP degradation.**

*A*, Representative western blots of APP and α-tubulin after 0.5 μM MG132 treatments. *B*, Quantification of mature and immature APP pooled from three experiments. Two-way ANOVA followed by Sidak’s multiple comparisons test.

**Figure S5. The effect of ammonia on lysosome morphology.**

*A*, Representative images after LysoTracker staining for lysosomes in control and NH_4_Cl-treated astrocytes. Nuclei were counterstained with DAPI (blue). n = 4. Scale bars = 20 μm. *B*, Fluorescence intensity quantification of LysoTracker-positive area of control and NH_4_Cl-treated astrocytes. Control n = 43 cells, NH_4_Cl n = 27 cells, Mann-Whitney’s U test, *****p*<0.0001. *C*, Cellular localization of APP (red), LAMP2 (green) and GFAP (white) in astrocytes with and without 72h NH_4_Cl treatments. Nuclei were counterstained with DAPI (blue). n = 3. Scale bars = 50 μm. *D*, Average Mander’s colocalization coefficients from 3 experiments. Control n = 43 cells, NH_4_Cl n = 34 cells, Mann-Whitney’s U test, *****p*<0.0001.

**Figure S6. The effect of ammonia on astrocytic ER.**

*A*, Cellular localization of APP (green), ER-selective dye ER-ID (red) and GFAP (white) in the presence or absence of NH_4_Cl (72h). Nuclei were counterstained with DAPI (blue). n = 3. Scale bars = 50 μm. *B*, Average Mander’s colocalization coefficients from 3 experiments. Control n = 38 cells, NH_4_Cl n = 36 cells, Unpaired *t* test, *****p*<0.0001. C, Cellular localization of APP (green), Golgi marker GM130 (red) and GFAP (white) in the presence or absence of NH_4_Cl (72h). Nuclei were counterstained with DAPI (blue). n = 3. Scale bars = 50 μm. *D*, Average Mander’s colocalization coefficients from 3 experiments. Control n = 41 cells, NH_4_Cl n = 32 cells, Unpaired *t* test. N.S. = not significant. *E*, Representative western blots of IRE1, phospho-IRE1 (Ser 724), and α-tubulin after 72h NH_4_Cl treatments. *F*, Quantification of total IRE1 and IRE1 phosphorylation (phospho IRE1) pooled from four experiments. Total IRE1 was normalized against α-tubulin and the phosphorylation of IRE1 was normalized against total IRE1. Unpaired *t* test. N.S. = not significant. *G*, Representative western blots of OASIS and α-tubulin after 72h NH_4_Cl treatments. *H*, Quantification of OASIS pooled from four experiments. Unpaired *t* test. N.S. = not significant.

**Figure S7. The effect of ammonia on the astrocytic production of Aβ42.**

*A*, Representative images of cultured astrocytes (GFAP) expressing Aβ42 in the ER (PDI) after 4h NH_4_Cl treatment with and without Dynasore and Brefeldin A. Nuclei were counterstained with DAPI (blue). n = 3. Scale bars = 50 μm. *B*, Representative images of GM130 staining in cultured astrocytes with and without Brefeldin A treatments. Nuclei were counterstained with DAPI (blue). n = 3. Scale bars = 20 μm.

**Figure S8. Hyperammonemia-mediated neuroinflammation in the hippocampal CA1 region of TAA mice.**

*A*, Visualization of reactive astrocytes in the hippocampal CA1 region of saline and TAA mice by GFAP immunostaining (red). All cells were counterstained for nuclei using DAPI (blue). Scale bars = 20 μm. n = 4. *B*, NeuN immunostaining in the hippocampal CA1 region of saline and TAA mice. Scale bars = 100 μm. n = 4. *C*, Expression of APP in the CA1 region of saline and TAA mice. Images showed GFAP (red), APP (green), and DAPI (blue). Scale bars = 20 μm (Low magnification images: 50 μm). n = 4. *D*, Expression of Aβ in the CA1 region of saline and TAA mice. Images showed GFAP (red), Aβ (green), and DAPI (blue). Scale bars = 20 μm. n = 3.
