# Supplementary figures and images for: Ammonia induces amyloidogenesis in astrocytes by promoting amyloid precursor protein translocation into the endoplasmic reticulum
Source: J Biol Chem. 2022 Apr 12;298(5):101933. doi: 10.1016/j.jbc.2022.101933 (PMC9117890; doi:10.1016/j.jbc.2022.101933)

Fig S1

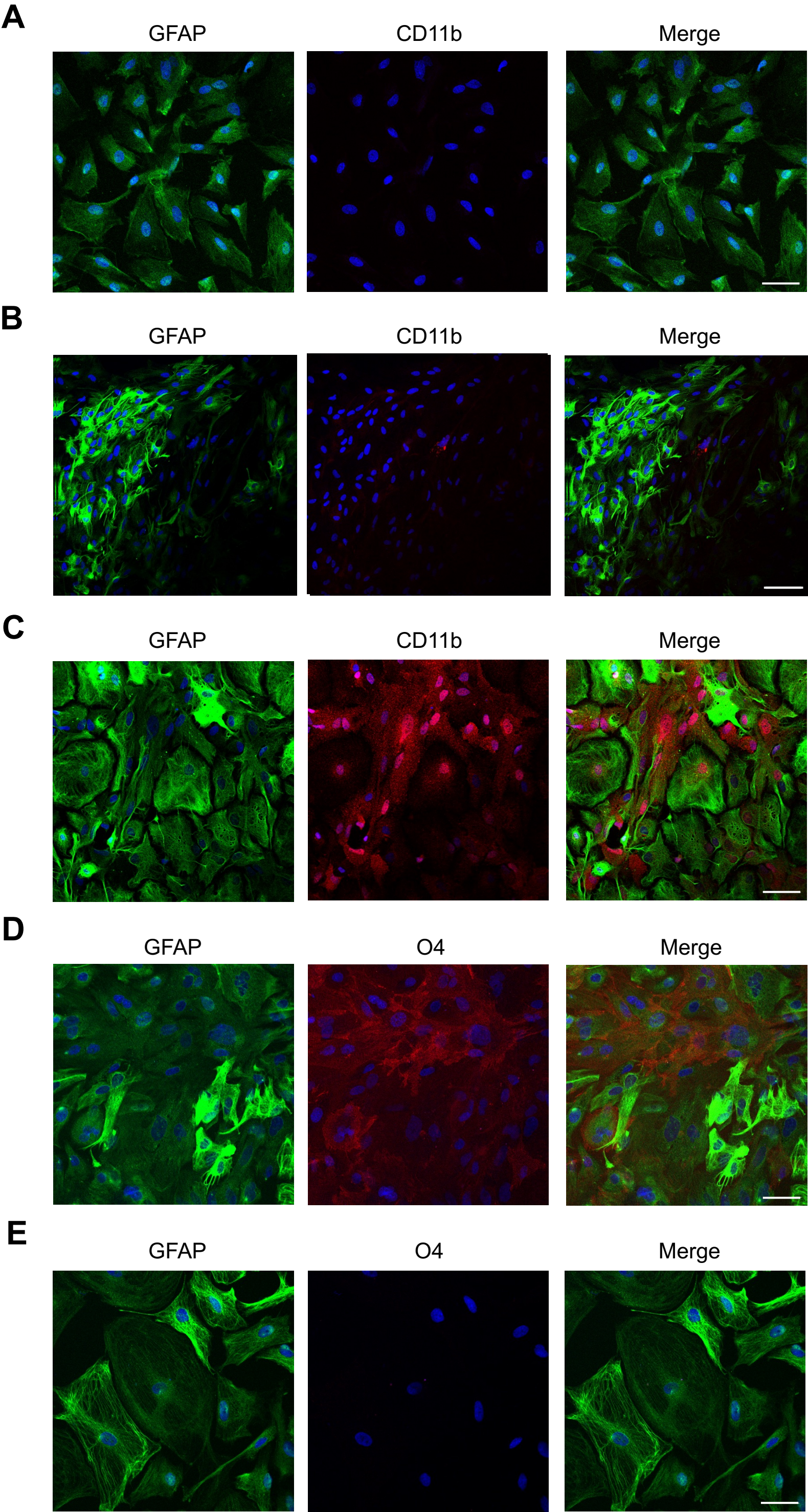

Supplement: Supplemental Figure S1 [file mmc2.pdf]

**Fig S2**

**A**

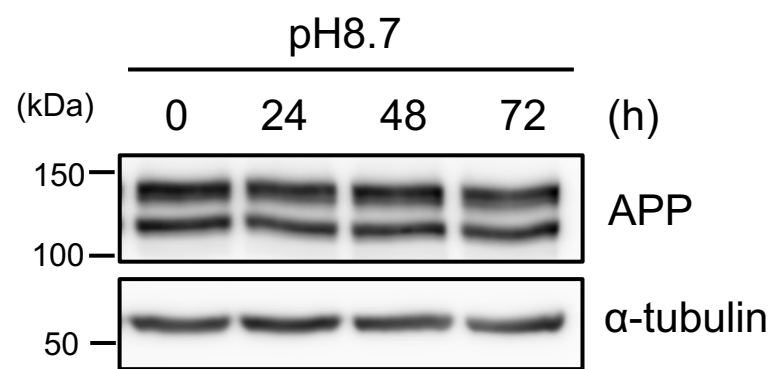

# B

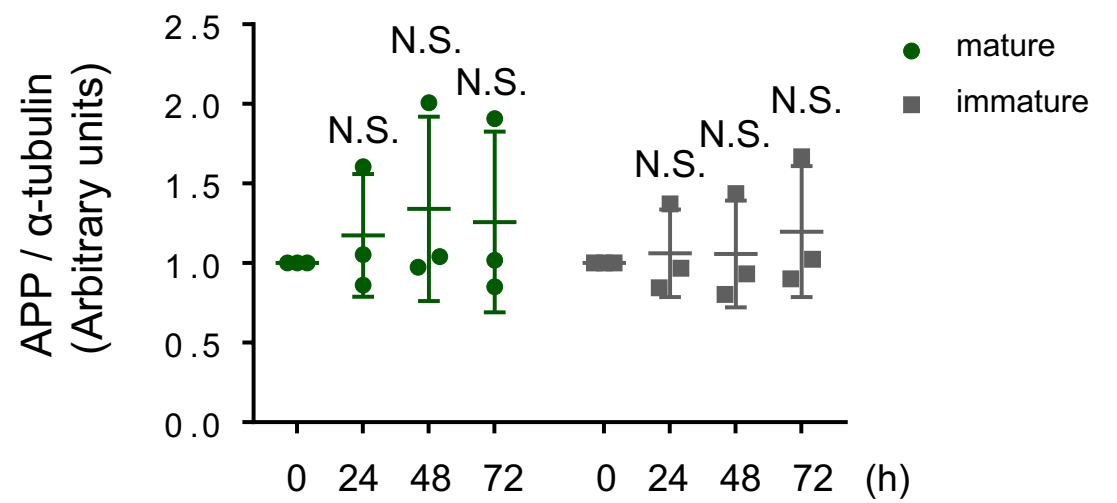

C

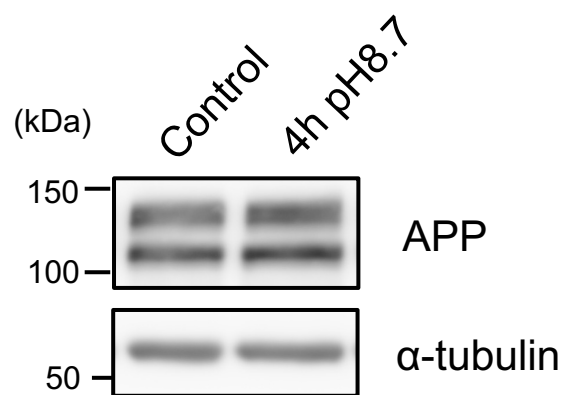

# D

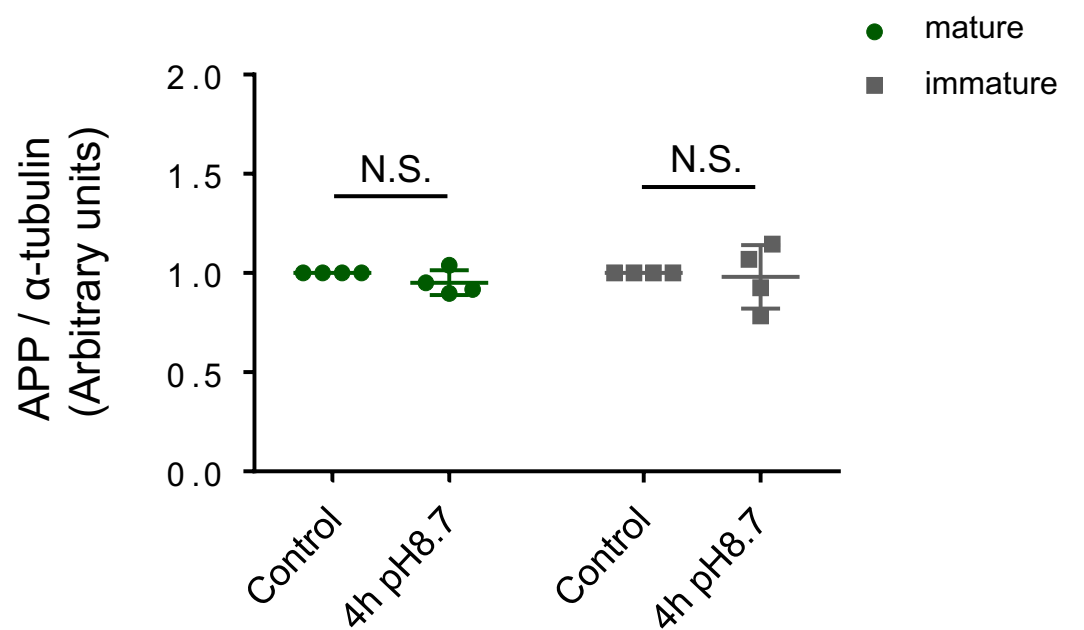

# E

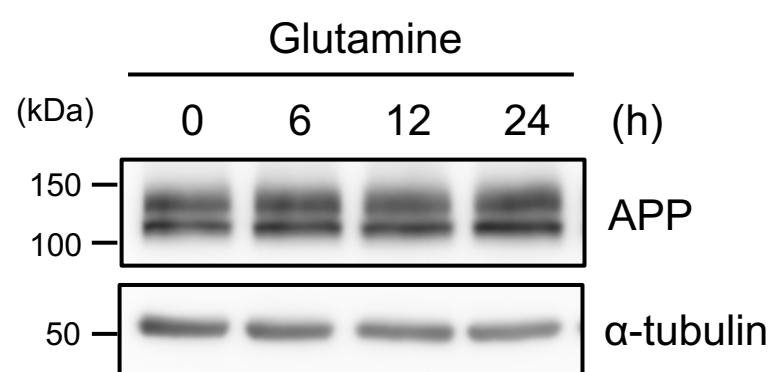

**F**

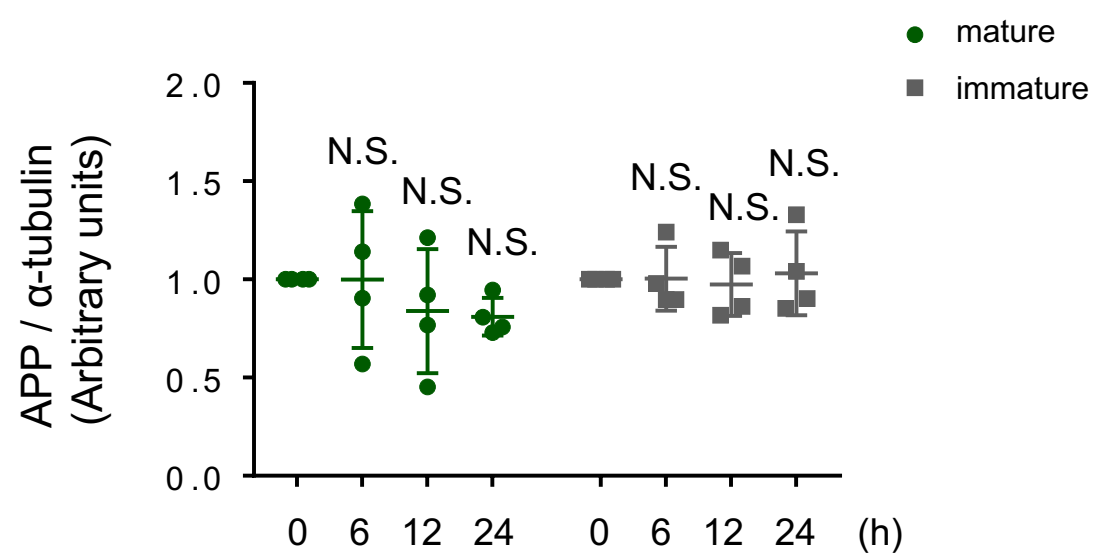

## G

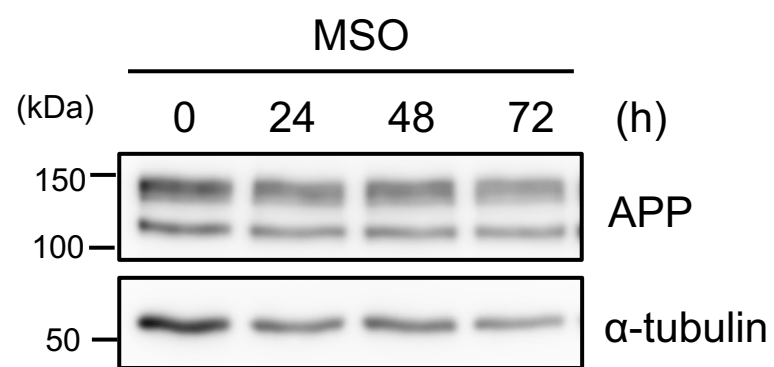

H

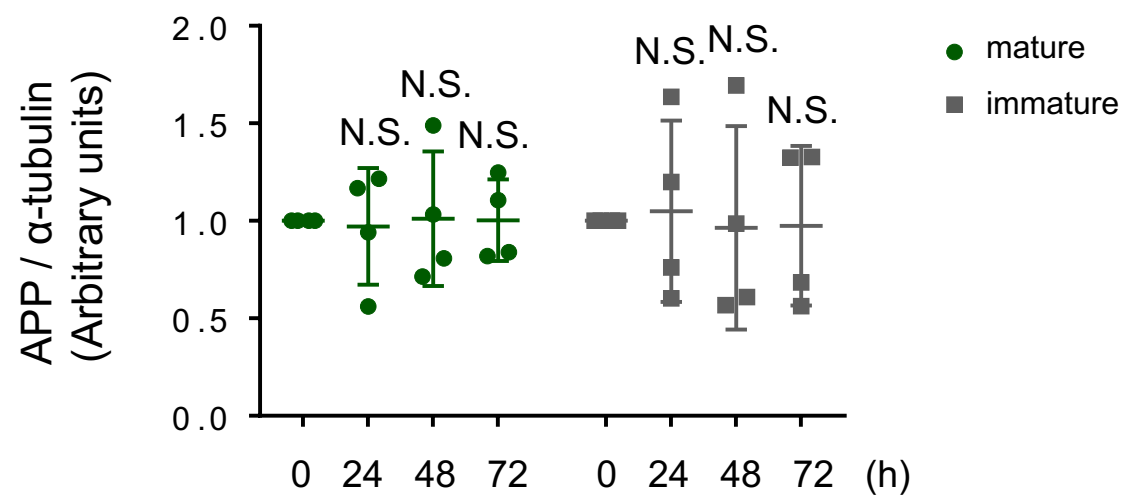

Supplement: Supplemental Figure S2 [file mmc3.pdf]

Fig S3

A

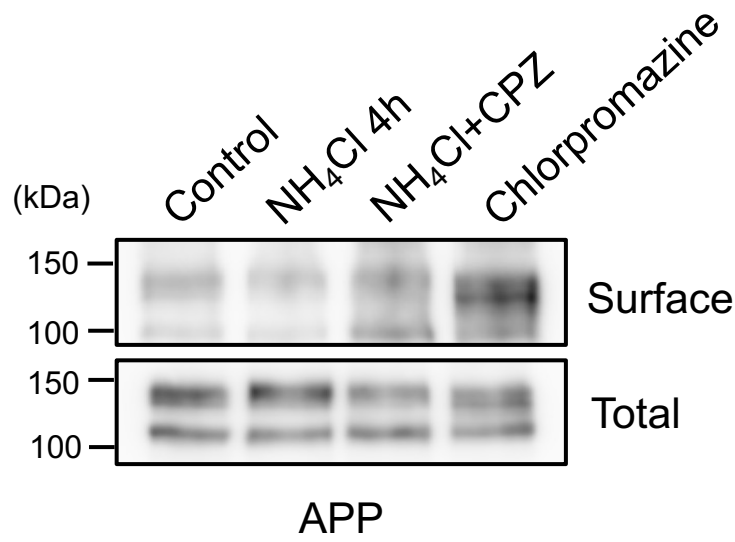

B

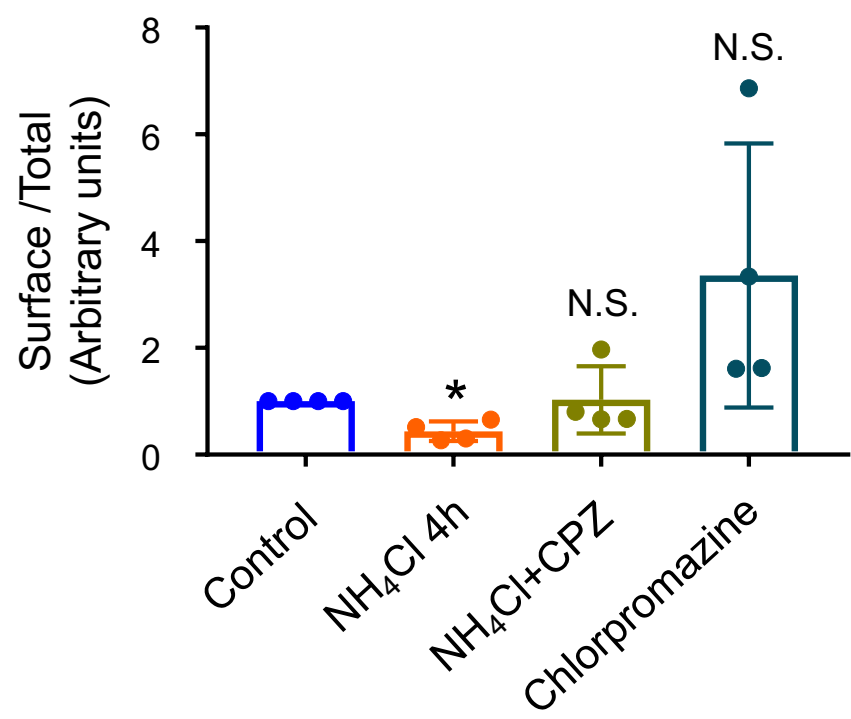

Supplement: Supplemental Figure S3 [file mmc4.pdf]

Fig S4

A

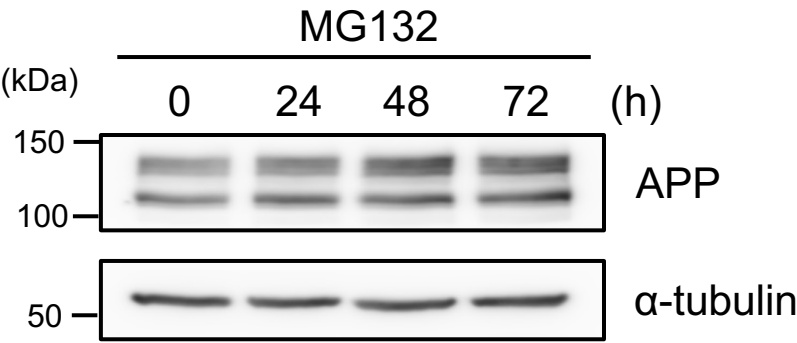

B

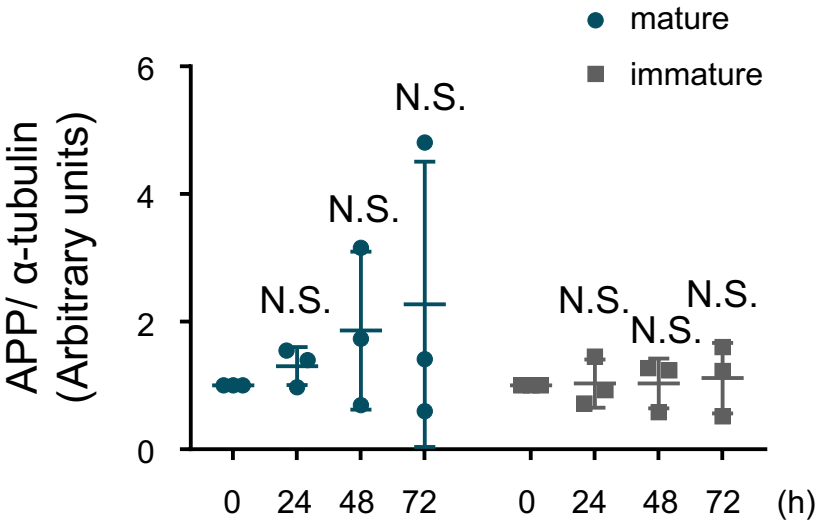

Supplement: Supplemental Figure S4 [file mmc5.pdf]

Fig S5

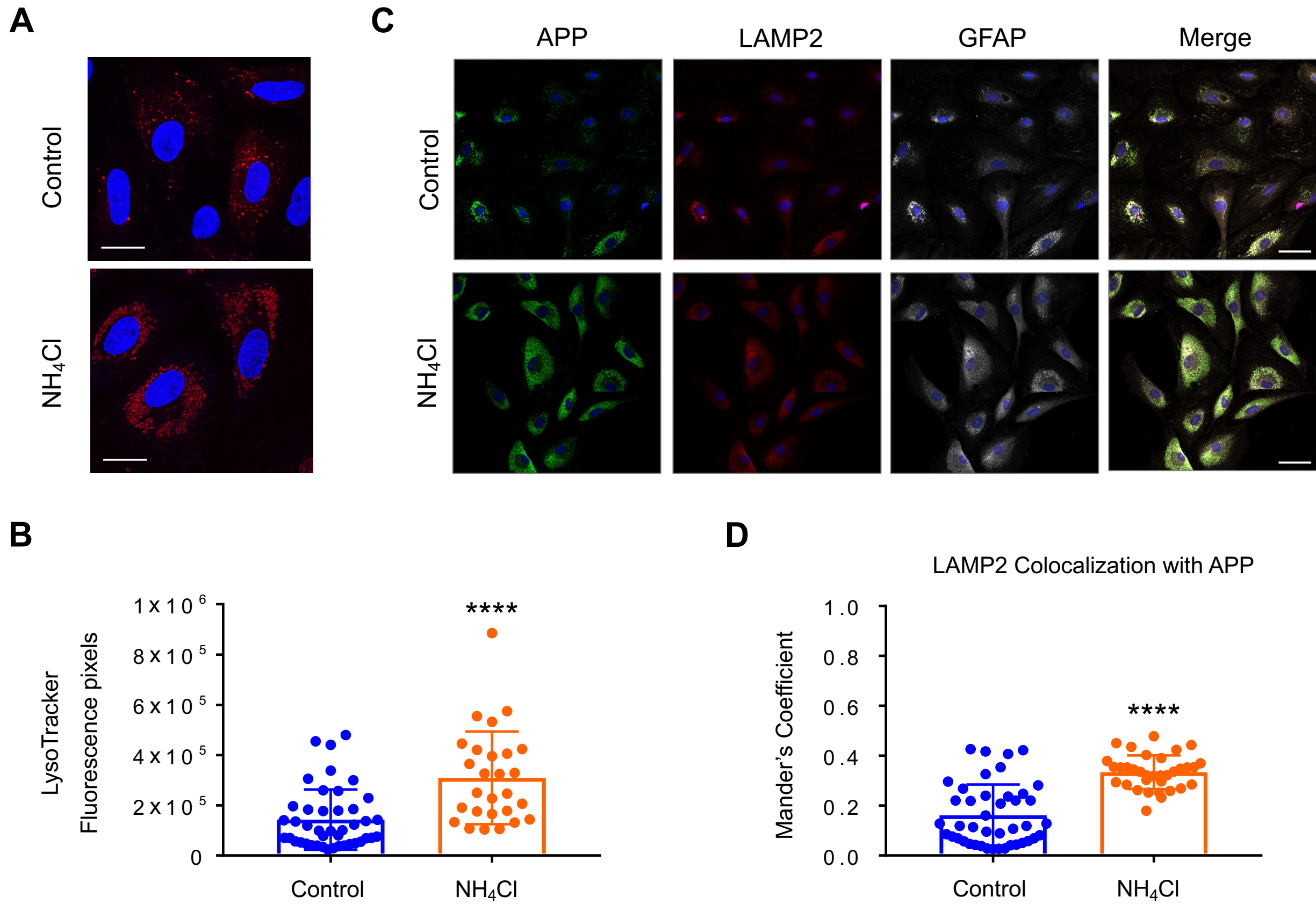

Supplement: Supplemental Figure S5 [file mmc6.pdf]

Fig S6

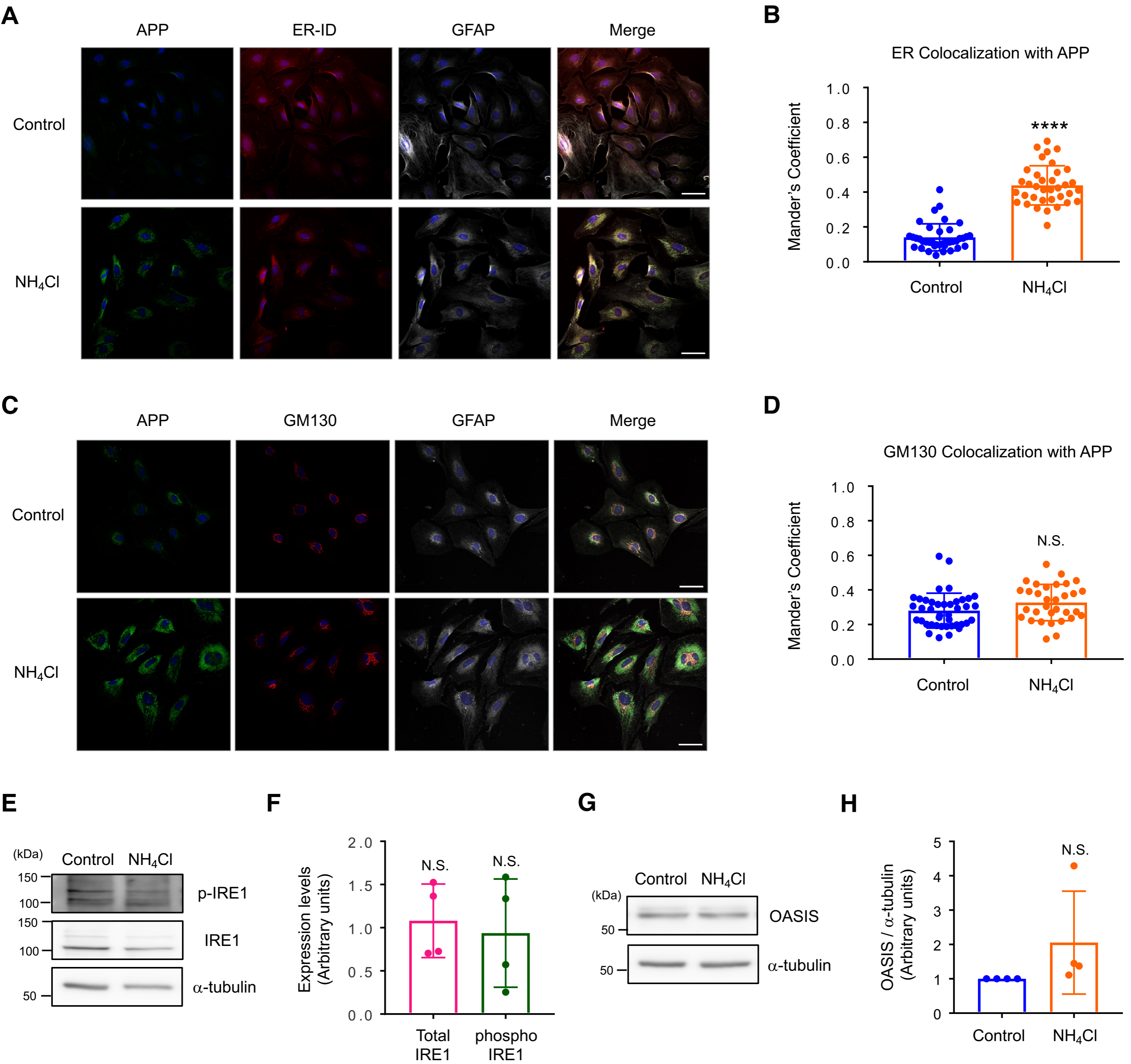

Supplement: Supplemental Figure S6 [file mmc7.pdf]

**Fig S7**

**A**

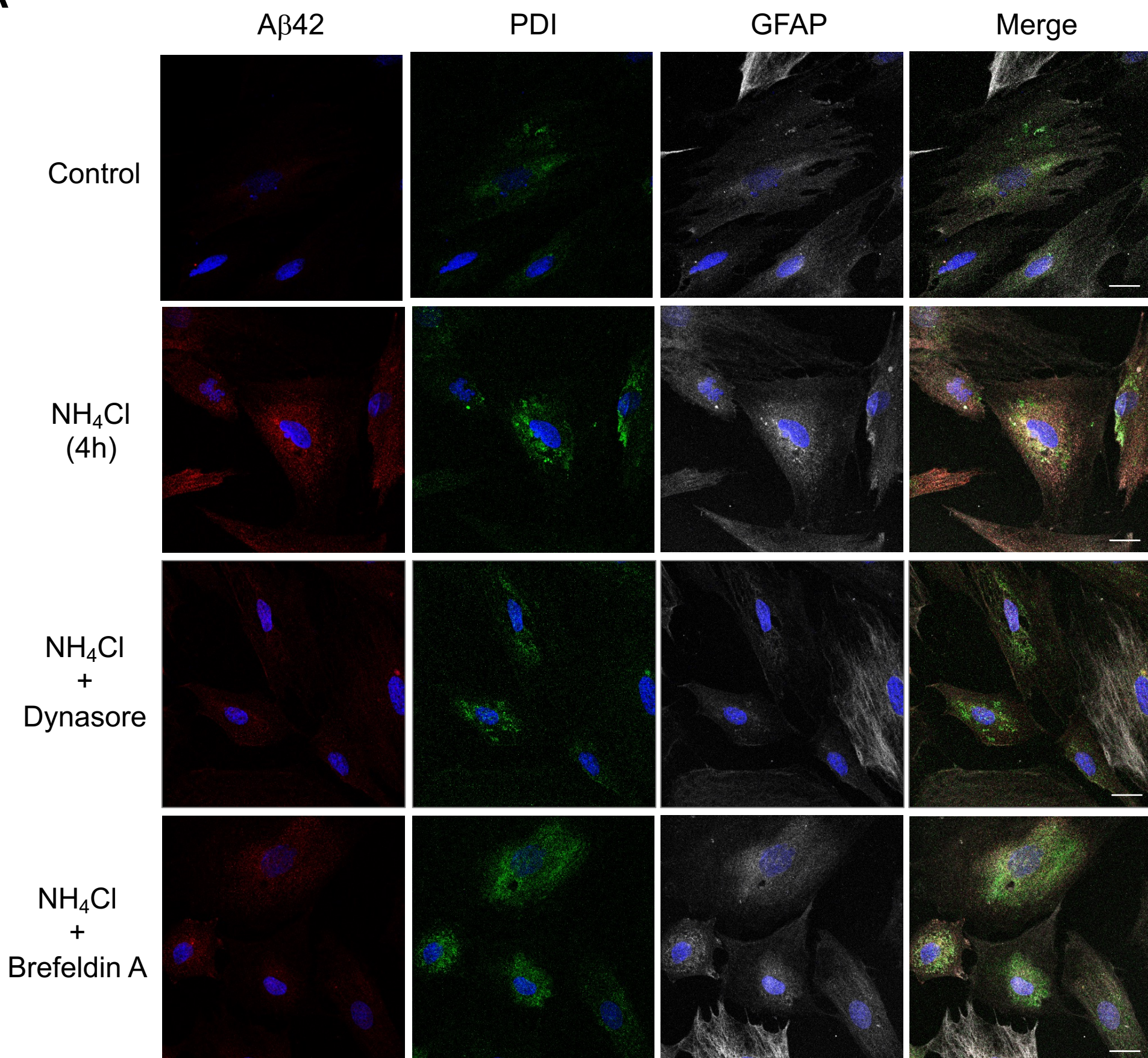

**B**

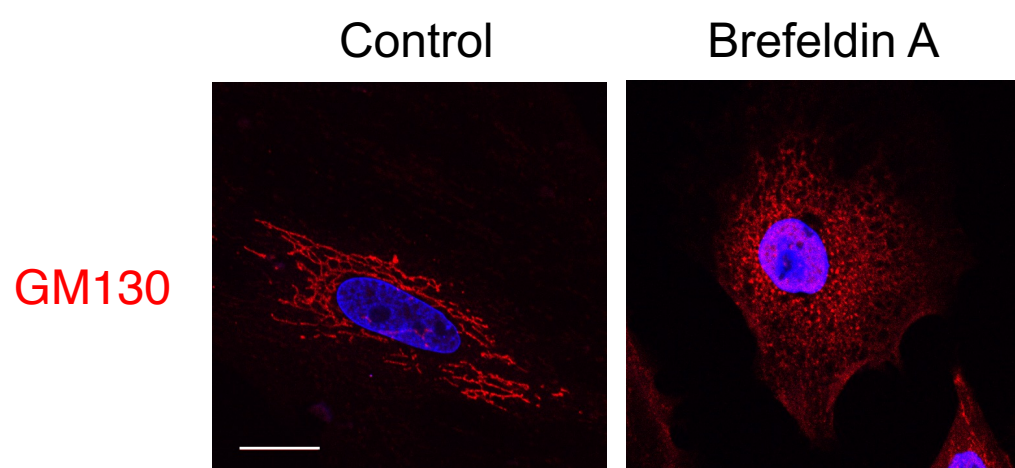

Supplement: Supplemental Figure S7 [file mmc8.pdf]

Fig S8

**A**

Saline

TAA

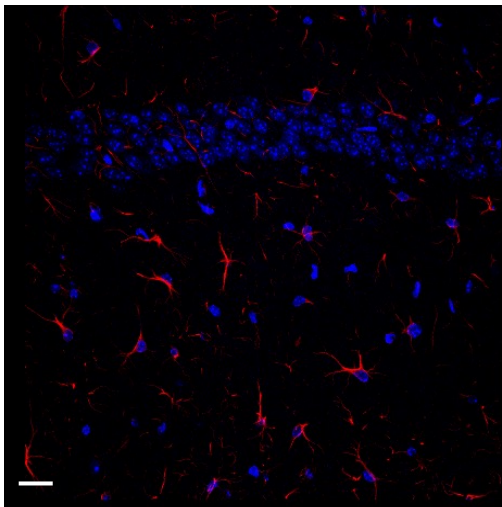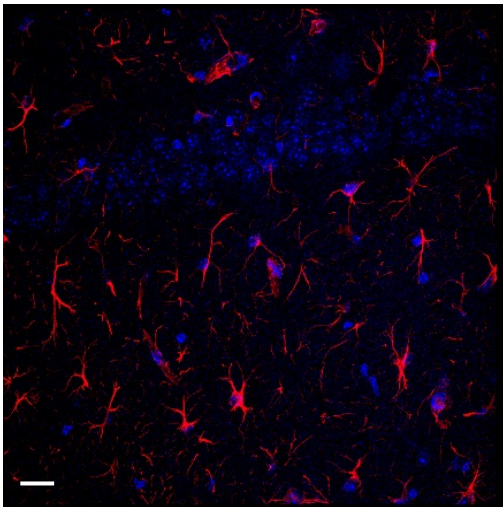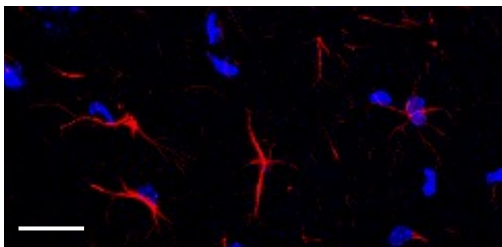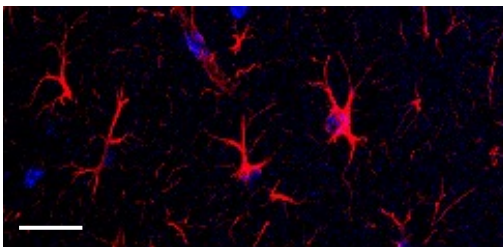

**B**

Saline

TAA

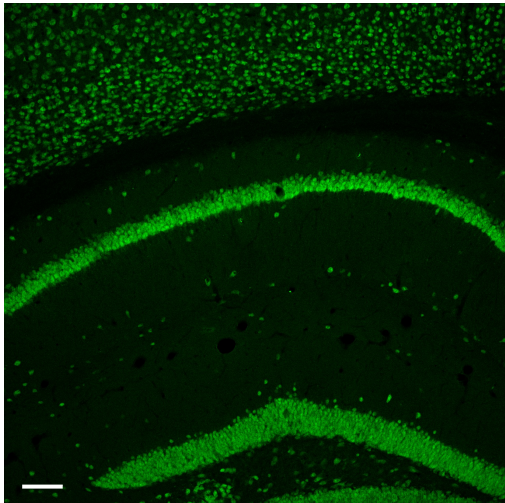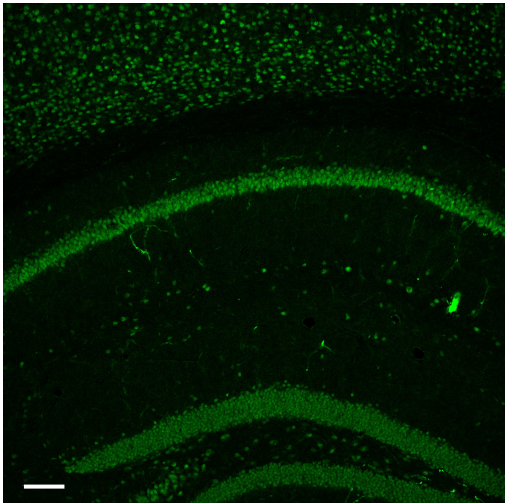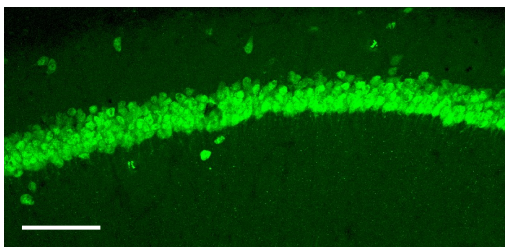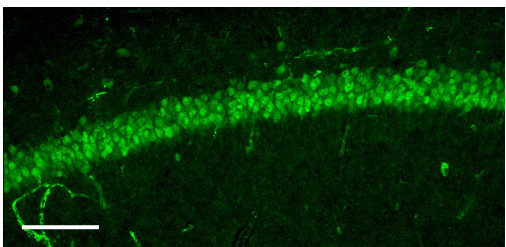

**C**

Saline

TAA

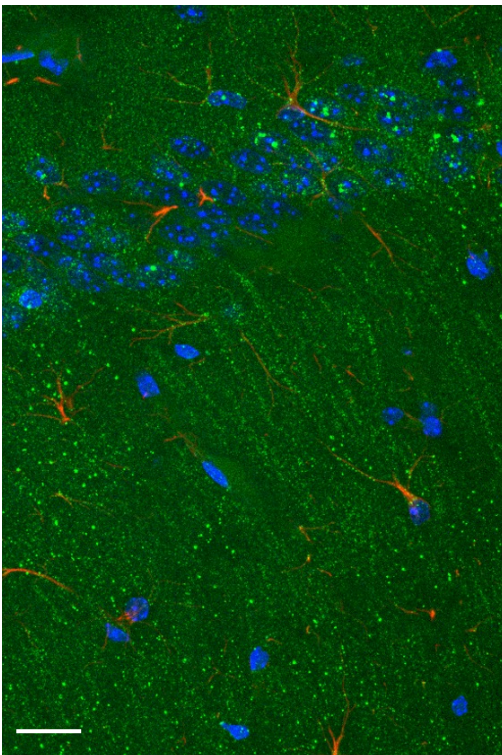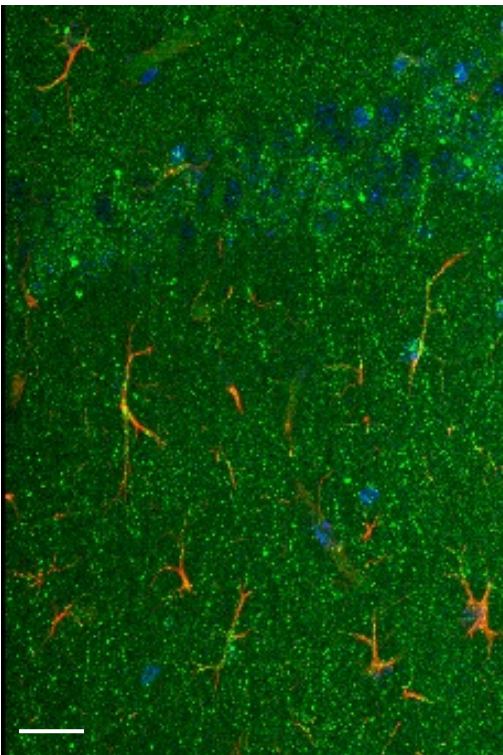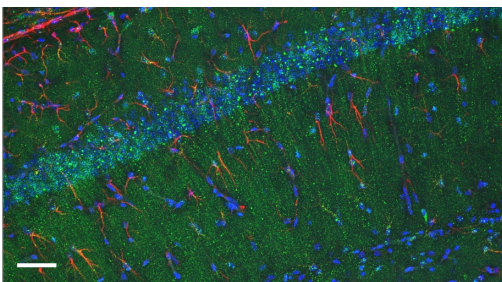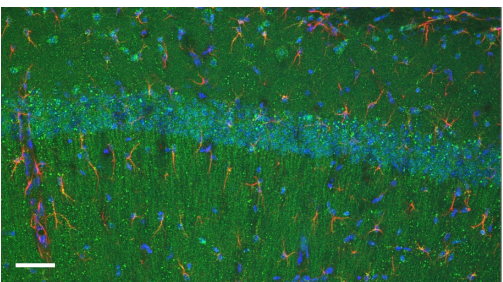

**D**

Saline

TAA

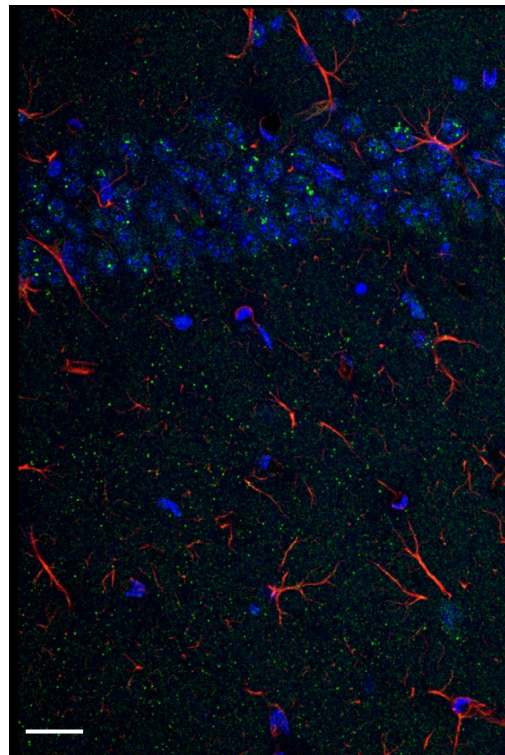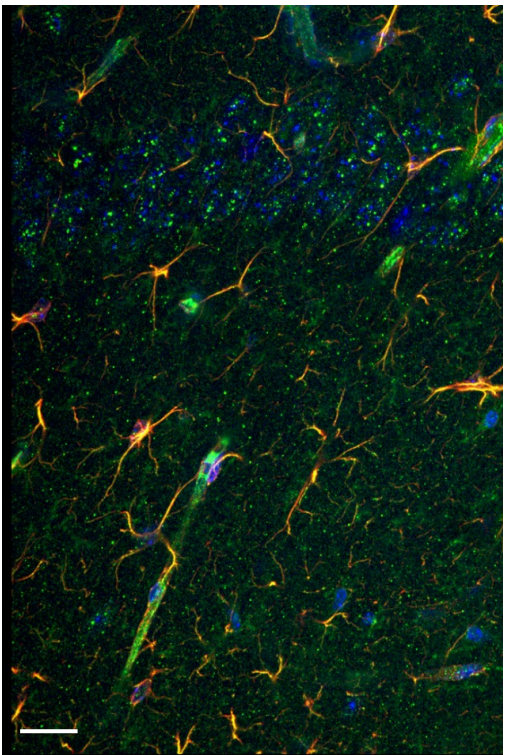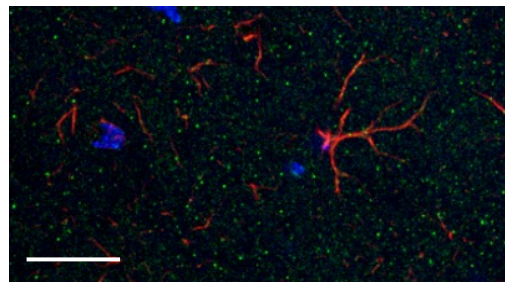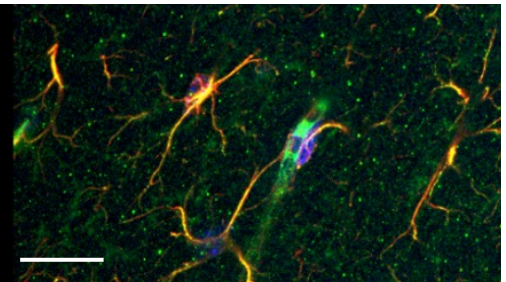

Supplement: Supplemental Figure S8 [file mmc9.pdf]
